# Harnessing the landscape of microbial culture media to predict new organism–media pairings

**DOI:** 10.1038/ncomms9493

**Published:** 2015-10-13

**Authors:** Matthew A. Oberhardt, Raphy Zarecki, Sabine Gronow, Elke Lang, Hans-Peter Klenk, Uri Gophna, Eytan Ruppin

**Affiliations:** 1 Blavatnik School of Computer Sciences and Sackler School of Medicine, Tel Aviv University, Tel Aviv 69978, Israel; 2 Department of Molecular Microbiology and Biotechnology, Faculty of Life Sciences, Tel Aviv University, Tel Aviv 69978, Israel; 3 Center for Bioinformatics and Computational Biology (CBCB), Department of Computer Science, and University of Maryland, Institute of Advanced Computer Science (UMIACS), University of Maryland, College Park, Maryland 20742, USA; 4 Leibniz Institute DSMZ—German Collection of Microorganisms and Cell Cultures, Braunschweig 38124, Germany; 5 School of Biology, Newcastle University, Newcastle upon Tyne NE1 7RU, UK

## Abstract

Culturing microorganisms is a critical step in understanding and utilizing microbial life. Here we map the landscape of existing culture media by extracting natural-language media recipes into a Known Media Database (KOMODO), which includes >18,000 strain–media combinations, >3300 media variants and compound concentrations (the entire collection of the Leibniz Institute DSMZ repository). Using KOMODO, we show that although media are usually tuned for individual strains using biologically common salts, trace metals and vitamins/cofactors are the most differentiating components between defined media of strains within a genus. We leverage KOMODO to predict new organism–media pairings using a transitivity property (74% growth in new *in vitro* experiments) and a phylogeny-based collaborative filtering tool (83% growth in new *in vitro* experiments and stronger growth on predicted well-scored versus poorly scored media). These resources are integrated into a web-based platform that predicts media given an organism's 16S rDNA sequence, facilitating future cultivation efforts.

## Abstract (web summary)

Culturing new microorganisms requires a great deal of experience, and trial and error. Here, the authors build a database of >3,300 culturing media recipes and >18,000 microbial species that allows the prediction of appropriate media recipes for the growth of new microbes based on their 16S rDNA sequences.

## Graphical abstract

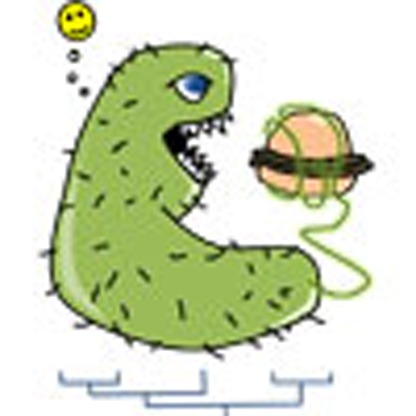

Culturing microorganisms is a classic microbiology challenge that is critical for tapping the biotechnological potential of microbial life. For example, a recent breakthrough in culturing of soil microbes enabled extraction of a new antibiotic compound that did not incur detectable resistance among tested pathogens, thus serving a critical need in human health^1^. Despite the very large time and effort spent in culturing organisms over the last century and a half, much of the process of developing of culture media is still strikingly similar to what it looked before the (gen)omics era (compare, for example, refs 2, 3, 4, 5, 6, from 1936, 1979, 2001, 2003 and 2012, respectively), and most microorganisms in nature still have not been cultured (∼99%, by classical estimates^7^). Best practices for culturing new organisms have been developed, and are embedded in guides such as Bergey's Manual of Systematic Bacteriology^8^. However, even with these best practices, the typical procedure for culturing a new microorganism still requires a great deal of experience and trial and error.

In recent years, some culturing efforts, particularly for difficult-to-culture organisms, have begun to include genome and pathway analysis^9^,^10^, as well as high-throughput technologies for determining microbial nutrient needs^11^. Instrumental in this work is pathway-based metabolic modelling, which encompasses many powerful tools for interrogating the metabolic capabilities of organisms^12^,^13^,^14^. Metagenomic sequencing technology, meanwhile, is now enabling the amassment of huge quantities of data about currently uncultured organisms. This confluence of technological, computational and theoretical advances marks a turning point and a challenge in ecological microbiology, as our data collection abilities now far surpass our ability to culture microbes. Integrating all of these areas will require fresh approaches to rapidly bring new organisms into culture. A logical starting point for this is to first catalogue the large current set of lab media that have been painstakingly and manually developed to date, and then to explore what insight these known media can give into predicting successful organism–media pairings.

Fortunately, a large collection of proven culture media exists in the Leibniz Institute DSMZ, a German non-profit centre that stores and disseminates microbes. The DSMZ repository (https://www.dsmz.de/?id=441) contains around 1,300 media (as well as many individualized variations) for around 23,000 microbial strains, encompassing the majority of culturing media in general use today. However, the DSMZ media are listed as recipes in non-standardized portable document format (PDF) files from which final compound concentrations can only be obtained via careful reading, cross-referencing, rearrangement and integration of strain-specific instructions. Codifying the exact nutritional compositions of these media is an undertaking of fundamental importance for microbiologists and systems biologists, but doing this requires collating and standardizing the component lists in these files, which is very tedious and a highly non-trivial task in itself.

In this work, we have integrated and codified these media documents into a relational database (KOMODO) that can be accessed computationally using Structured Query Language, enabling an analysis of broad features and trends in proven lab media. Notably, the database we have built is an order of magnitude larger than a previous effort to build a known media database^15^: our database contains 18,049 species, 3,335 media and 20,824 organism–media pairings, whereas the previous database included 208 species, 461 media and 765 organism–media pairings. Our compilation has enabled the systematic study of the majority of growth media for the first time, revealing patterns and principles determining whether organisms tend to grow on media. We mine this database in the context of microbial phylogeny to explore which nutrients are most commonly used in media across the tree of life, and which ones are most differentiating between close species. Next, we develop a phylogeny-based predictor of new organism–media pairings, which enables successful prediction of new organism–media pairings among cultured organisms. We provide this resource in an online searchable database and a tool that predicts media for any bacteria or archaea, given a 16S rDNA sequence or National Center for Biotechnology Information (NCBI) taxon ID (the tool can be found at: http://delta-tomcat-vm.cs.tau.ac.il:40678/komodo/default.htm).

## Results

### An overview of KOMODO, the known-media database

A large collection of media for bacteria and archaea is publically available through the collection of strains and media at the DSMZ (https://www.dsmz.de/?id=441), but these media are listed in non-standardized PDF files that cannot be computationally accessed. Putting these media into a usable database form required extensive and non-trivial work, parsing, merging and organizing, as well as handling cross-references between media and submedia compound mixtures such as ‘trace element solutions,' which could be detailed and referenced from any DSMZ medium. This was achieved by extensive manual curation followed by an automated pipeline to import the data, and finally several validation checks against ‘gold-standard' data sets that were curated semi-manually. This pipeline is depicted in the flowchart in Supplementary Fig. 1, and is thoroughly described in the Methods.

**Figure 1:**
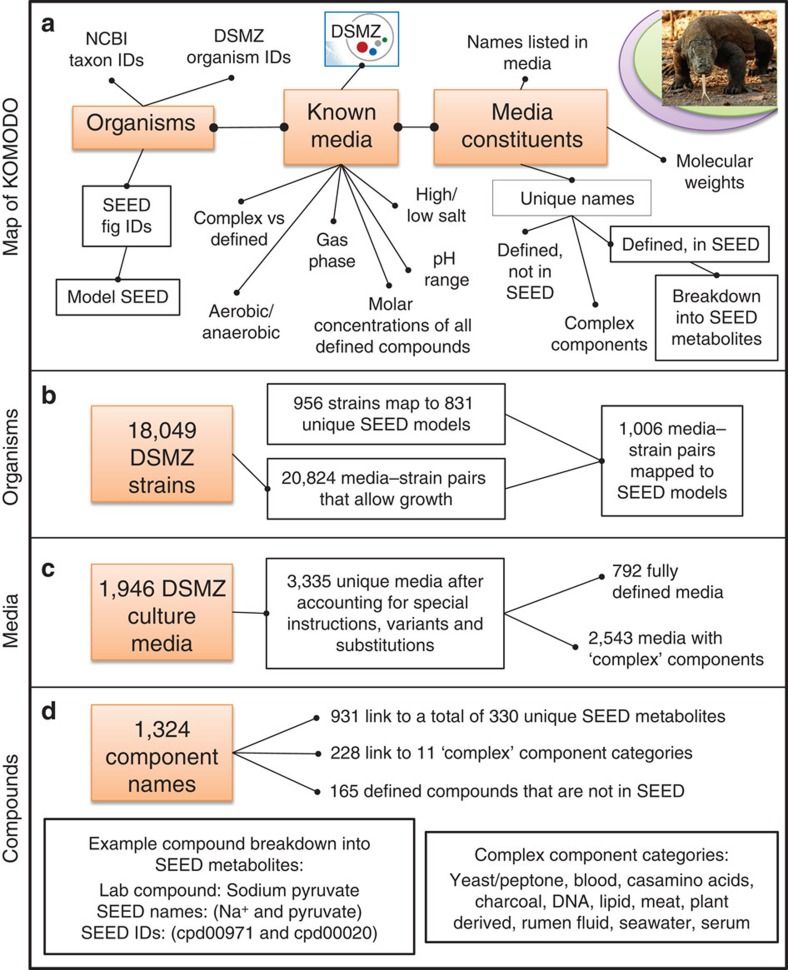
Schematic of KOMODO, the Known Media Database. The contents of KOMODO are shown. (**a**) A map of the structure of the database, showing how major tables and information points connect. (**b**–**d**) Numbers of organisms, media and nutritional components present in the database. SEED refers to the Model SEED database^16^; see KOMODO website and Methods for more details.

The result of the work is the Known Media Database (KOMODO), a database of microbial culture media that encompasses almost the entire DSMZ collection. The database includes 3,335 media variants (expanded from an initial ∼1,300 because of special instructions and substitutions), 1,324 unique metabolic component names composing the media, 18,049 microbial strains and 20,824 media–strain pairings. The basic structure of KOMODO is shown in Fig. 1. Components in KOMODO were decomposed when possible into chemical names from Model SEED, a large, consistent systems biology and genome annotation database^16^. This was done to eliminate degeneracies in component names, as a service to the bioinformatics and metagenomics communities that use SEED, and especially to enable integration and comparison of KOMODO media with genome-scale metabolic modelling efforts in the future (along the lines of ref. 17). KOMODO has been made available for browsing by users at: http://delta-tomcat-vm.cs.tau.ac.il:40678/komodo/default.htm. It has also been leveraged to create an online tool that enables users to input a bacterial or archaeal 16S rDNA sequence or an NCBI taxon ID and in turn get predicted media that the organism can grow on (this tool is based on GROWREC, as described below, and is provided on the same website).

### KOMODO reveals global patterns of media and compound usage

**Figure 2:**
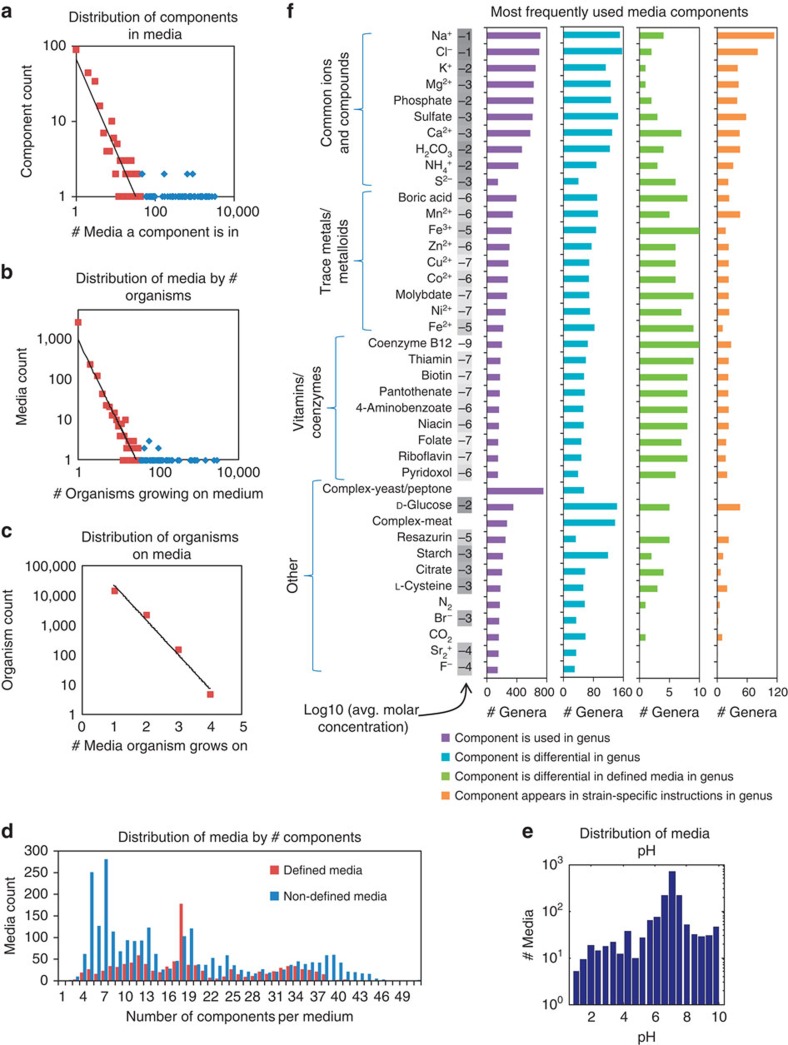
Large-scale properties of known media. (**a**) Distributions of components in media. This includes both defined and complex/undefined components, where undefined components are grouped into their complex categories and each category present in a medium is counted as one component. (**b**) Distributions of media by the number of organisms that grow on them. (**c**) Distributions of the number of media that organisms grow on. (**d**) Distribution of media by the number of components within them. (**e**) Distribution of pH values of known media. Red squares in **a** and **b** denote the bins used for the power law fit. (**f**) The 40 most frequently used media components across genera. Ions listed here were typically added to media as salts, which we assume completely dissociate in solution (for example, MgCl_2_ becomes Mg^2+^ and Cl^−^). Components are broken into four groups: biologically common ions/compounds, trace metals/metalloids, vitamins/coenzymes and other. Within each group, components are listed in order of their frequency of usage across genera, from most to least. Left of the bar graphs is a list of average concentrations of each component in media across KOMODO, listed in units of log10(molar concentration). A component is ‘differential' in a genus if it appears in media for some strains in that genus but not others.

Typical patterns of media usage and composition in KOMODO can reveal fundamental trends in microbial nutrition, as well as gaps and investigator biases. To gain an overview of these relationships, we built histograms of component, media and organism distributions across the database (Fig. 2a–d). We observed that component usage across media, and media usage across organisms both follow power-laws, which suggests a ‘rich gets richer' structure to the assignment of phenotypes and component usages (Fig. 2a,b). This structure might reflect converging nutrient requirements among organisms, the preferential way in which ‘successful' media are selected for organisms by human investigators, or both.

In contrast, the distribution of the number of media an organism is listed to grow on does not follow a power law, but rather follows an exponentially decaying distribution with the largest number of established media per organism in DSMZ being 4 (Fig. 2c). This pairing of organisms with media is from the internal databank of DSMZ, and clearly reflects heavy under-sampling because of an investigator bias, as researchers would typically seek only one or a few media per organism, rather than exhaustively seeking all media that an organism might grow on. Indeed, only 0.04% of potential organism–media pairings are listed in the database (and only positive growth phenotypes are listed); it is highly likely that many more pairings could enable growth, as supported by the *in vitro* success of our novel growth predictions (see section below). We also examined how many components both defined and non-defined media contain (with each complex category present in a medium considered to be one ‘component'—see Fig. 2d). There is a large range of media sizes (that is, the number of distinct components making up a medium) even among fully defined media, reflecting the variable inclusion of trace element and vitamin mixtures aside from likely differences in biological needs of bacteria. Few truly minimal media, that is, those containing the smallest number of distinct nutrients possible while still enabling a microorganism to grow, exist in the DSMZ database (or are listed as such), reflecting the typical goal of culturing efforts to get microorganisms into culture quickly and easily, rather than to determine their minimal nutritional requirements.

The pH values of media range from 0.8 to 10.1, with 76% of media having a pH between 6 and 8, 15% below 6 and only 9% above 8 (Fig. 2e). It is notable that alkaline media are fewer and closer to neutral pH than the acidic media. This is despite the large diversity of high pH-tolerating organisms in, for example, soda lakes, which have pHs of up to around 12 and are among the photosynthetically most productive environments on the Earth^18^. High pH-tolerant organisms might represent a gap in the ranges of investigated organisms. However, the distribution of media also may reflect neutralizing/acidifying effects of CO_2_ on natural macro-environments, whereby environments without a replenishing source of alkalinity tend to drift down the pH scale, and thus are indeed less common than more neutral or acidic environments^18^. In addition to pH, we examined salt usage across media. Principle component analysis of the media-by-component concentration matrix revealed that Na^+^ and Cl^−^ ions are the most dominant components separating all media, as they are frequently used and are often present at high concentration values (see Supplementary Figs 2 and 3 and Supplementary Note 2 for details).

To gain a more complete picture of component usage across the tree of life, we examined which components are used in the media of the most genera (Fig. 2f, left bar graph). We observed that the most frequent components are biologically common ions/salts that are usually present as macronutrients (1–100 mM), followed by trace metal elements and vitamins (which are present in 0.1–10 μM and 1–1,000 nM ranges, respectively; see concentrations bar on the left of Fig. 2f). Also frequent across genera are some complex media components (peptone and meat extract) and the carbon sources (glucose and starch). Some of these components might be added to many media but are not selective between related organisms. To better understand which components are actually differentiating between similar species, we checked the number of genera in which a component is present in media for some species/strains but not for others. We did this first taking into account all media, and then only considering fully defined media (Fig. 2f, middle two bar graphs). Strikingly, when considering only fully defined media, the components that are differential across the most genera are trace metals and vitamins/coenzymes, despite their being less commonly used across genera than the common ions/salts. This signal was likely hidden when also including non-defined media as complex components such as meat extract provide many of these trace components at once. Supporting this observation, the ‘complex-meat' category is a highly differentiating component across genera when considering all media—see second bar graph in Fig. 2f.

This analysis points to trace compounds such as metal ions and cofactors as key ingredients to consider when trying to grow new species from within cultured genera, a principle that has been noted recently in trying to culture as-yet-uncultured genera as well^1^,^19^. For comparison, we also extracted from KOMODO all instances where a nutritional ingredient is specifically added, removed or has its concentration changed from a base medium in order to grow a given strain, and assessed the number of genera in which each component is altered this way (Fig. 2f, right bar graph). The most frequently altered compounds in this way are the biologically common ions/salts, followed by trace metals and vitamins. This lends further evidence that these trace components play key roles in differentiating growth between close species, and thus should be considered in future media design.

Beyond these analyses, we examine broad trends in compound usage across phyla at different taxonomic levels. Heat maps of enrichment of different taxonomic groups for media components can be found in Supplementary Figs 4–8 and Supplementary Note 3.

### Media usage follows phylogenetic and ecological trends

**Figure 3:**
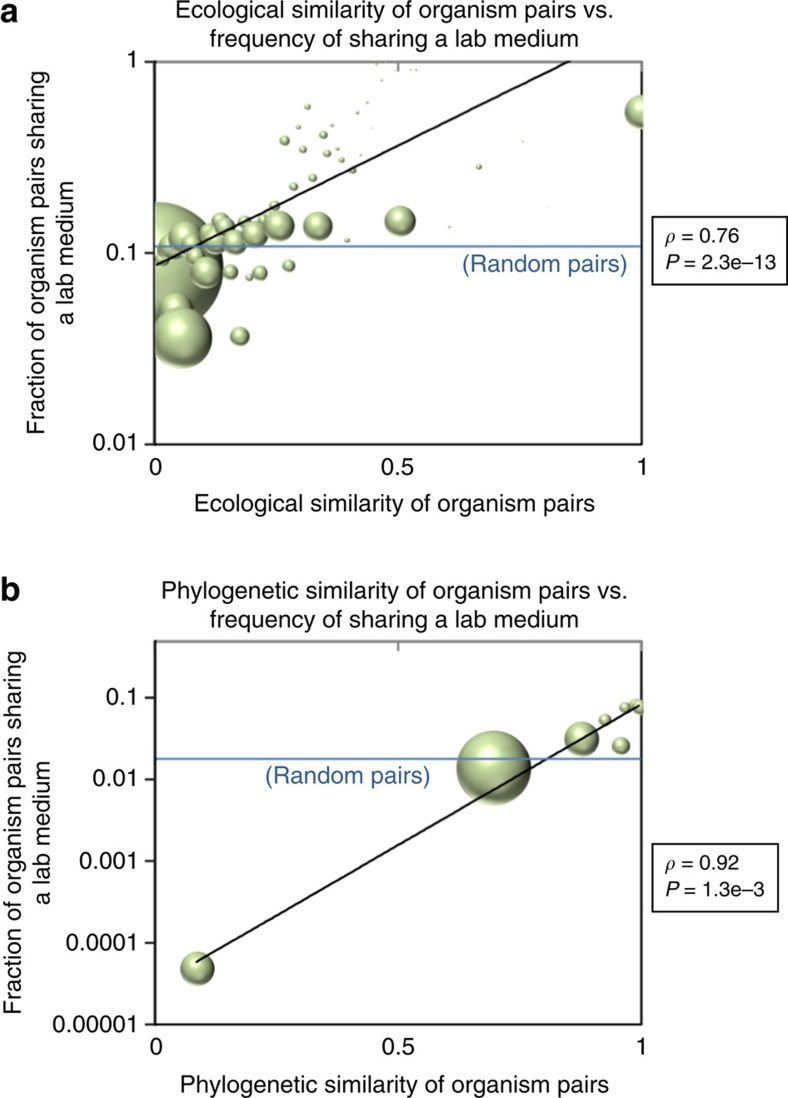
Media usage is correlated with ecological and phylogenetic similarity. The (**a**) ecological and (**b**) phylogenetic distances between pairs of species are plotted versus the fraction of species pairs within each ecological or phylogenetic distance bin that share at least one DSMZ medium. Bubble areas are scaled to the number of organism pairs in each bin. The fraction of random organism pairs of any ecological/phylogenetic distance sharing a lab medium is shown by the horizontal blue line, for reference. Distances are determined by a Jaccard metric of ecological co-growth in Greengenes database (ecological) or by subtree distance (phylogenetic; Methods).

An implicit assumption that investigators make when trying to cultivate new microorganisms is that the best medium to start with is one from a phylogenetic or ecological neighbour. Despite its apparent logic, this assumption has not, to our knowledge, been rigorously tested and validated. To do this, we mapped organisms in DSMZ to operational taxonomic units in Greengenes ecological data as clustered into environments (see Methods for details; clustering in ref. 20), and also to taxonomic classifications from the Interactive Tree of Life project (Itol^21^). We find that, indeed, the likelihood that two organisms share at least one lab medium strongly correlates with both their ecological and phylogenetic similarity (see Fig. 3; *ρ*=0.76, *P*=2.3e−13, and *ρ*=0.92, *P*=1.3e−3, respectively, for ecological and phylogenetic similarities, as determined by cohabitation Jaccard index (ecological) or inverse subtree count in the iTOL taxonomic tree (phylogenetic); see Methods for details). This indicates that phylogenetic and ecological closeness are good heuristics for determining the likelihood that two organisms have successfully been grown in the same lab medium. Indeed, we show later that this is not just descriptive of what has been done in the past, but that it holds a signal that can be used predictively for deriving new successful organism–media pairings. Importantly, the fractions of organism pairs sharing lab media listed in Fig. 3 are likely underestimates, as the organism-by-media matrix in KOMODO is highly underpopulated (see previous section). This observation is indeed upheld when we perform new growth experiments, as most of our predictions (which were not listed previously in KOMODO) yield growth.

In addition to exact media matches as just described, we tested whether these associations would also hold for partial matches between lab media. We considered two organisms to have a partial medium match if a comparison of the best matching media of the two organisms (or of the sets formed from the unions of components from all of their listed media) exceeded a specified similarity threshold (see Methods for details). Indeed, over a range of thresholds and with all combinations of these metrics, the correlations between media similarity and ecological or phylogenetic relatedness are maintained (see Supplementary Fig. 13). These associations not only indicate that ecological or phylogenetic data may be helpful in guiding culturing of organisms on known lab media, but, importantly, that these data may be usable for determining likely subsets of media to include for a given organism when developing novel media formulations.

### Predicting new organism–media pairings via transitivity

**Figure 4:**
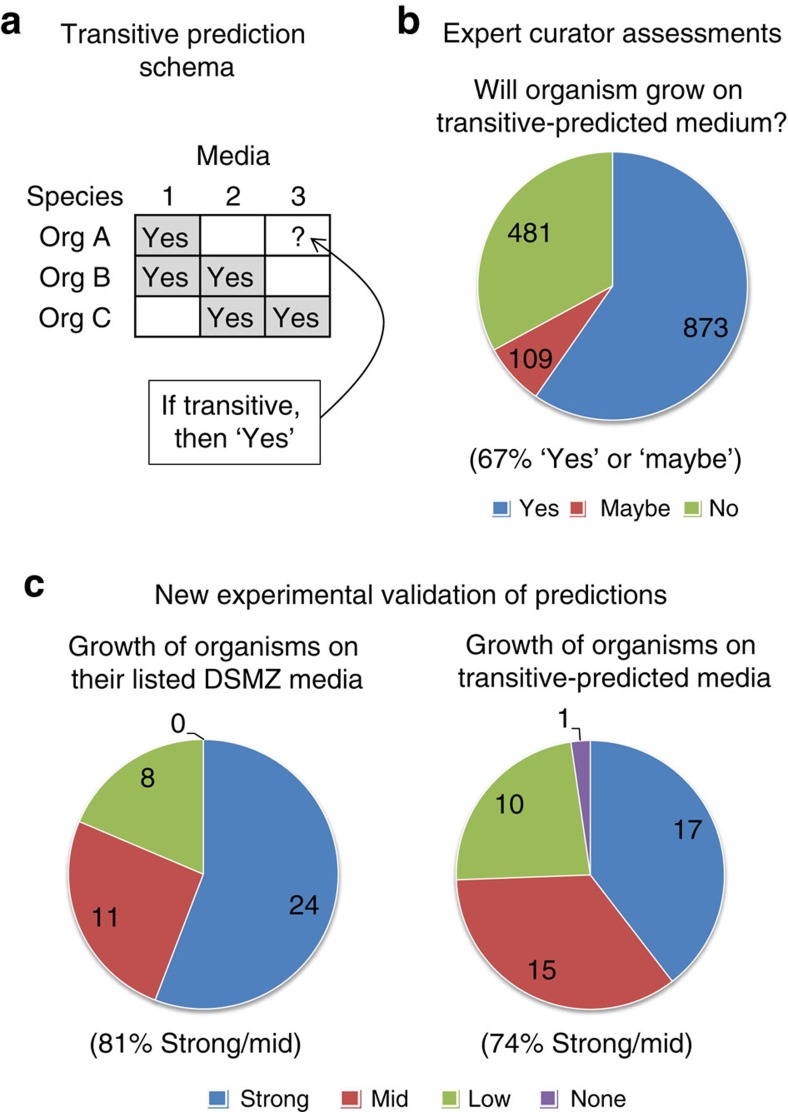
Transitive media predictions. Organism–media pairings are predicted based on an observed transitivity heuristic following a schema shown in (**a**). Organisms orgA and orgB share a medium (M1), organisms orgB and orgC grow on M2 and the third organism orgC grows on medium 3; we then predict, based on transitivity, that orgA will grow on medium 3. (**b**) Distribution of expert DSMZ curator opinions on whether organisms will grow in transitive-predicted media (full opinion descriptions are provided in Supplementary Data 1). (**c**) Pie charts that represent the number of growth phenotypes observed for organisms grown *in vitro* on their listed lab media (left) and for the same organisms grown on newly predicted media (right). Numbers in the pie charts show the number of organism–medium pairs tested.

A fundamental property in logic and math is the potential transitivity of a given relation **R**; that is, if **R** is transitive, then (A**R**B and B**R**C) implies that (A**R**C). To better understand the known patterns of microbial growth in KOMODO, we asked whether the property ‘organism sharing of media' is transitive. To test this, we searched the KOMODO data for growth media patterns involving three organisms A, B and C, in which it is given that A and B grow together on a certain medium (m1), B and C grow together on another medium (m2) and C grows on a third medium (m3; see Fig. 4a). Given this pattern and assuming transitivity, we predicted that organism A would be more likely than a randomly chosen organism to grow on m3 (see Methods for details). Out of 1,000 such tests, we identified 694 positive (that is, documented growth) instances of transitivity versus only 1 positive case among the 1,000 randomly selected organisms. This result strongly indicates that media preferences are indeed transitive (binomial *P*<1e−186).

The significant under-sampling of bacterial growth media per organism that is manifested in KOMODO means that a large number of potential organism–media pairings may be viable, but have simply never been tested *in vitro*. We therefore used the transitivity identified above to predict a total of 15,147 new organism–media pairings that may be viable which, if true, would nearly double the number of organism–media pairings in the database (from an initial 20,840). Interestingly, when including all of the new transitive-predicted phenotypes in the database, we observed that the distribution of organisms on media now does follow a power-law, as opposed to the exponential distribution seen originally (compare Supplementary Fig. 14c and Fig. 2c). Using this expanded database to re-evaluate how phylogenetic or ecological distance affects the chance for two organisms to share a lab medium (analogous to Fig. 3) yielded a significant correlation versus ecological distance but only borderline-significance against phylogenetic distance, indicating the limits of this method (see Supplementary Fig. 9 and Supplementary Note 4). We additionally tested how often non-defined or ‘rich' media (see later sections for ‘richness' definition) participate in transitive associations. We found that the most common and accurate transitive predictions are for organisms known to grow on rich media, but that these predictions can be based on transitive associations linking through media of any richness (see Supplementary Note 5 for details).

### Experimental validation of transitivity predictions

To test whether organism–medium pairings predicted via transitivity are valid, we performed the following two-step process. First, we asked expert curators at DSMZ to assess the reasonableness of a large set of more than 1,000 predictions. Four experts participated in this test. Given lists of organism–medium pair predictions, they were asked to label the reasonableness of the predictions, and to give comments if they had particular insights. We classified their responses into three categories: yes, no and maybe/indeterminate. In all, they confirmed 64% of predictions they assessed (that is, they said ‘yes' for 873 out of 1,354 predictions, with 109 ‘maybes'—see Fig. 4b).

Next, we chose a subset of 43 predictions to test experimentally in lab. For this, we chose organism–media pairings with varying curator confidences (24 predicted to yield growth, and 19 unsure or predicted not to). We found that a remarkable 74% of predicted pairings yielded strong or medium growth, with positive growth phenotypes found among all of the curator-predicted classes (strong or medium growth was found in: 20 of 24 predicted to grow; 3 of 6 predicted not to grow; 3 of 7 listed as ‘maybes' and 6 of 6 not previously assessed by the curators). This success rate may be further appreciated by noting that the percentage of mid-to-high growth of the same organisms when grown on their standard listed DSMZ media is only slightly higher, reaching a level of 81%, albeit with a higher percentage of ‘strong' versus ‘mid' growth (Fig. 4c). These experiments demonstrate our ability to harness growth media transitivity to predict many new organism–media pairings that are almost as good as existing ones.

### Predicting media usage via collaborative filtering

**Figure 5:**
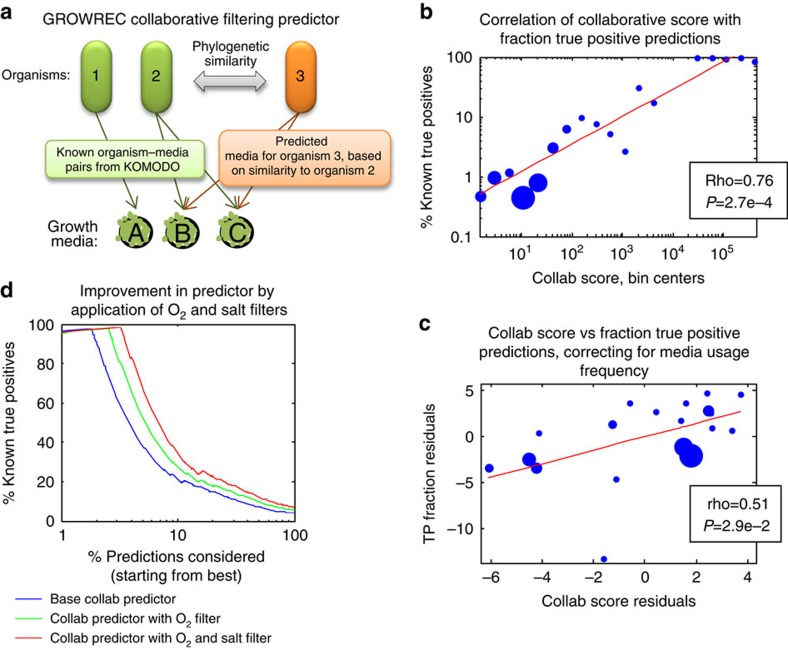
Collaborative filtering predicts media usage. (**a**) The concept of collaborative filtering. In brief, the media preferences for a new organism (org3) are predicted based on known preferences of phylogenetically similar organisms (here, org2). (**b**) Circles represent bins per collaborative score, with diameters proportional to the number of organism–media pairs per bin. Collaborative scores correlate with the true positive fraction (that is, the number of organism–media pairings known in the actual DSMZ database). (**c**) The partial correlation of collaborative (=collab) score versus true positive fraction, corrected for media usage frequency. (**d**) The true positive percentages of collaborative filtering predictions from GROWREC are presented with the base predictor, and with oxygen and/or salt filters added on. The *x* axis shows the % of organism–media pair predictions considered (starting from the one with the highest collaborative score and taking predictions in descending order of collaborative score), and the *y* axis shows the percentage of predicted organism–media pairs within a given set that are known true positives (that is, are already listed in KOMODO).

The associations we observed between phylogenetic (or ecological) distance and likelihood of two organisms sharing a lab medium, as well as the observation of transitivity of growth on lab media, suggest a new way to predict new organism–medium pairings. To leverage these observed relationships towards this goal, we developed a collaborative filtering medium recommendation system, which proceeds in two steps. Given an input ‘test' organism for which we aim to predict growth media, we first select a set of organisms from within KOMODO that are phylogenetically (or ecologically) close to the ‘test' organism (which is not required to be in KOMODO). Next, we integrate the known medium preferences of those organisms into a ‘collaborative score' (or ‘collab score') that indicates which media the test organism is likely to grow on (see Fig. 5a and Methods for details). The phylogeny-based predictor was used in all following analyses, unless specifically stated otherwise; the ‘closeness' threshold was set at a phylogenetic subtree distance of 0.04, as this yielded the most accurate results (see Supplementary Note 6 for a sensitivity analysis of this threshold).

In all, our phylogenetic-based collaborative filtering predictor (hereafter called GROWREC) recommended 47,060 organism–medium pairings. (A higher threshold could yield a score for any organism–medium pairing, but lower specificity (for example, see Supplementary Fig. 12c–e for details).) Of these predictions, 1,768 (4%) were ‘true positive' organism–medium pairings (that is, those already present in the DSMZ database), a percent extremely unlikely by chance (*P*=5.4e−49 in empirical permutation test when compared with a ‘null' predictor based solely on the relative abundance of media and organisms in KOMODO—see Methods for details). It is important to note that GROWREC predictions take into account the media preferences of phylogenetic neighbours of an organism, but do not take into account any known media associations of the organism itself. Therefore, true positive media pairings predicted for an organism are not circular, but rather represent the success of a leave-one-out test. Remarkably, the collaborative score correlates extremely strongly with the rate of such ‘true positive' organism–medium pairings (Fig. 5b; ρ=0.76, *P*=2.7e−4 in Spearman test, where fractions of true positives are plotted per bin of collaborative scores). The collaborative score also strongly correlates with medium usage frequency from the database (Supplementary Fig. 15a; rho=0.87, *P*<3e−5 also binning by collaborative score). Therefore, we used partial correlations as controls and found that the frequency of true positives correlates with the collaborative score even when correcting for media usage frequency (Fig. 5c; ρ =0.51, *P*=2.9e−2 in a partial Spearman test).

Among the most important factors determining microbial growth on a medium is the salt content (as revealed via principle component analysis; see Supplementary Fig. 2) and the presence or absence of oxygen. To further refine GROWREC, we classified organisms into salt and oxygen usage groups (for example, high salt, low salt and so on) based on observed patterns of growth on DSMZ media, and imposed filters on GROWREC predictions to eliminate pairings of high-salt organisms with low-salt media, and so on (see Methods and Supplementary Note 7 for full details of the analysis). This significantly increased the accuracy and quality of our predictions (for example, total true positives increased from 3.8 to 6.8%, with 20,357 (44%) predictions eliminated without losing true positives). To display this improvement, we show a plot of GROWREC accuracy versus the percent of results considered (starting from those with the best scores), pre- and post-filtering (Fig. 5d). It can be seen from the plot that filtering allows consideration of nearly twice the number of results, while keeping the same expected accuracy.

### GROWREC predicts levels of key nutritional factors in media

**Figure 6:**
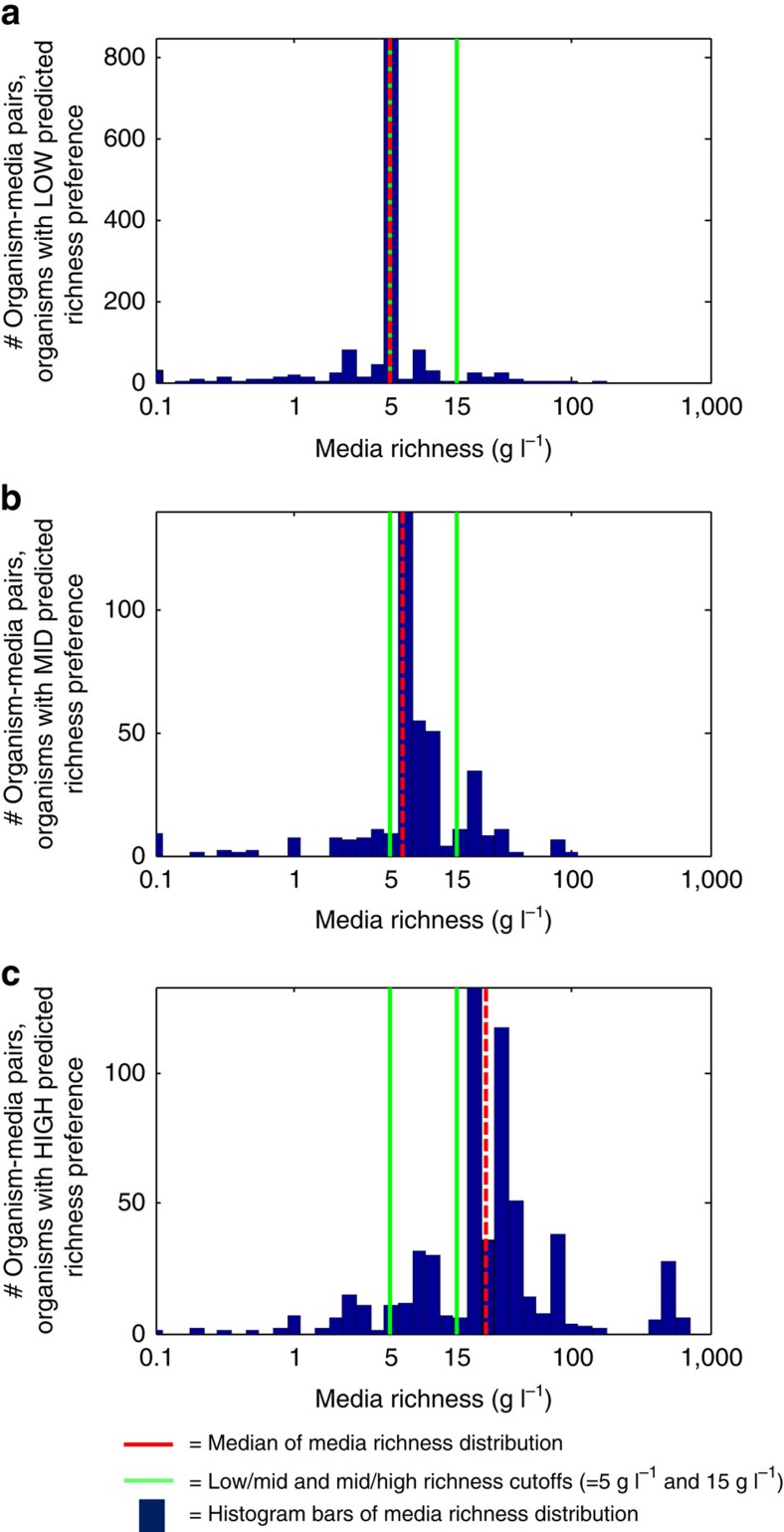
Predicted richness preferences of organisms reflect richness of confirmed growth media. Organisms are split into three groups based on their predicted richness preferences: (**a**) low, (**b**) medium and (**c**) high. We then plot histograms of the ‘richness' of media paired in the DSMZ repository with organisms within each group. The red vertical line in each plot denotes the median of the distribution, and the green lines denote cutoffs of low/medium and medium/high richness (5 and 15 g l^−1^, respectively).

Aside from predicting full media (as just reported), we were interested in using GROWREC to predict strain preferences for key nutritional features, as such a method could then be used in principle to design novel media. We tested this concept on the feature of media ‘richness,' which should be matched with strain richness preference in order to obtain optimal growth. We define ‘richness' as a weighted sum of complex components and carbon compounds that captures the total amount of nutritional carbon available for consumption; see Supplementary Table 1 and Supplementary Data 4 for lists of rich components. Namely, we predicted each organism's richness preference by taking a sum of the richnesses of media predicted for it by GROWREC, weighted by the respective collaborative scores (see Methods for details). This predictor attained high accuracies of 66.4 and 79.6% against manually curated richness preferences and a gold-standard richness preference data set from KOMODO (see Methods and Supplementary Note 8 for details). Predicted richness preferences were strongly consistent with the richness of media that the organisms are known to grow on, indicating the marked usefulness of our classifier (see Fig. 6). Importantly, the predictions for a given organism did not take into account its own known richness preferences, so testing against these known preferences is not circular. This also means that the richness predictor may be applied to organisms not already listed in KOMODO (in the same manner that GROWREC can—see Methods for details).

This methodology can be used in principle to predict or optimize key media components for difficult-to-culture organisms, or ones that have never been cultured before. To assess this task, we identified a set of 70 bacteria that were cultivated from soil in a 2002 study using minimal agar medium with xylan, a polysaccharide containing repeats of xylose, as the sole substrate^22^. None of the bacteria had been cultured before, and none are currently in the DSMZ collection. Based on 16S rDNA sequences provided in the source paper, we used GROWREC to predict the most likely media for these organisms to grow on. We then examined the predicted media for 17 different major component categories (the categories were previously defined—see Supplementary Data 4 for details). We found that among these component categories, which include, for example, sugars, amino acids, co-enzymes and salts/ions, the category that showed up most often in high versus low scored media was polysaccharide (*P*=2.0e−11 in ranksum test of GROWREC scores for media containing versus not containing any polysaccharides). The category ‘sugar' (which includes mono- and di-saccharides, and some sugar alcohols) showed the reverse trend, that is, that media not containing sugar had the highest GROWREC scores (*P*=1.9e−4 in ranksum test for sugars). Thus, GROWREC selectively identified the key component class needed to extract these organisms in otherwise minimal media, based only on 16S rDNA data. In future growth experiments, GROWREC might be used in a similar way to determine the critical component classes needed for growing new organisms, assuming that like these organisms, they have simple (that is, non-exotic) but specific nutrient requirements.

### Experimental validation of GROWREC

As before, we employed a two-step testing process to evaluate the collaborative filtering predictions. First, we asked expert curators at DSMZ to assess the reasonableness of predictions from GROWREC after we had removed results that did not pass our salt and oxygen filters (see Fig. 5d). Each curator commented on a subset of organism–medium pairings from our predictions, starting from those with the highest collab scores. Six curators in all gave assessments, over a total of 681 new organism–medium pair predictions. We next assigned a ‘yes', ‘no' or ‘maybe/unsure' to each assessment, as we had done in the analysis of transitive predictions. We found that positive curator assessments tended to have higher collaborative scores than negative ones (*P*=1.9e−47 or *P*=2.5e−11 in ranksum tests for collaborative scores being higher in high or mid/high versus mid/low or low curator assessments, respectively).

**Figure 7:**
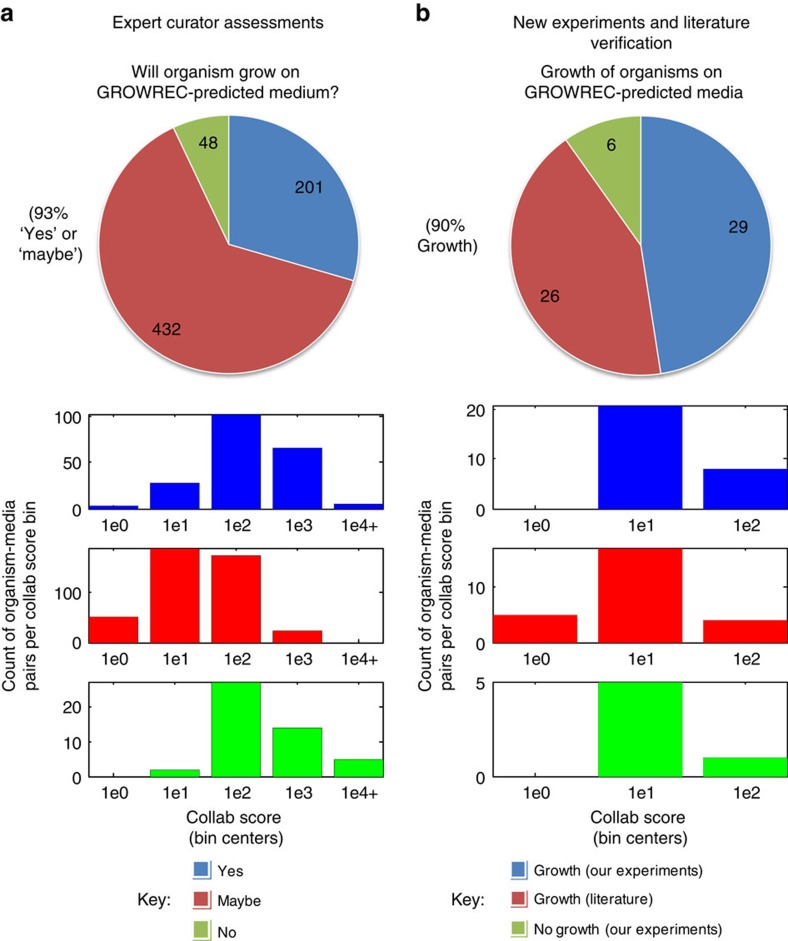
Curator assessments and experimental validation of GROWREC predictions. (**a**) Expert curator opinions on the goodness of GROWREC predictions. (**b**) Results from our *in vitro* growth experiments or found in literature verifying top GROWREC predictions. Histograms in **a** and **b** represent the distributions of collab scores for the org-medium pairs assessed, and are coloured the same way as the pie charts. Numbers in the pie charts denote how many org-medium pairs were tested for growth.

Of the 681 newly predicted organism–media pairings that the curators were able to assess, they marked 93% with either ‘yes' or ‘maybe.' This is remarkable, considering that most of these GROWREC predictions had collaborative scores corresponding with much lower expected success rates (overall, we expected only 4% success, based on extrapolations from our true positive rates versus collab scores curve shown in Fig. 5b). The distribution of collaborative scores assessed, and the breakdown into ‘yes', ‘no' and ‘maybe' categories, also brings up an unexpected observation that even many of the low-scored predictions had much higher than expected accuracy. For example, the curators deemed 9.6% of predictions within the lowest third of collaborative scores that they assessed to be ‘yes', as opposed to an expected rate of 1.2% (that is, based on Fig. 5b—see the histogram in Fig. 7a). This suggests that our ability to predict viable media is much higher than that suggested from our previous analysis of true positive percentages, and that the successes may also range to much lower than expected collaborative scores.

In a second testing step, we chose 61 predicted organism–media pairs to study further. These pairings were taken from the top range of our predictions (see the histogram in Fig. 7b for the range of collab scores, and see Methods for a detailed explanation of how pairs were selected for testing), and fell into the likely (18) or uncertain (43) curator opinion categories. We were able to verify 26 of these predictions by literature search or in-house DSMZ data that had not been included in KOMODO. For the 35 predictions remaining, we conducted new *in vitro* growth experiments, from which we obtained growth in 29 cases (11 of which had been classed by curators as ‘maybe'). Taken in full, 90% of these 61 organism–medium pairings either yielded strong or medium growth in new experiments, or were shown in previous reports to be viable (Fig. 7b). The projected accuracy of the assessed predictions based on their collaborative scores (using the collab-score-to-TP-ratio mapping from Fig. 5b, as before) is 5%, again significantly lower than the 90% accuracy obtained. These results indicate that GROWREC predictions even with mid-range collaborative scores have much higher than expected accuracies.

It was possible that the high percentage of positive growth phenotypes we observed was due to a general ability of organisms, particularly aerobic heterotrophs, to grow on any of the common lab media. It was important, therefore, to test growth of organisms on the predicted media as compared with a baseline of poorly predicted media, and to assess the difference. We therefore chose a set of 40 ‘good' organism–medium pairs and also a set of 40 ‘bad' organism–medium pairs as predicted by GROWREC (the ‘bad' pairs were predicted using a permissive phylogeny cutoff—see Methods for details). Importantly, we chose the ‘good' and ‘bad' pairings using the same exact set of organisms and media, but merely swapping them around to form optimal good or bad combinations. To avoid trivial obstacles to growth, we did not include any organisms or media that grow in high salt or in anaerobic environments. The analysis included 36 bacterial strains each belonging to a different Genus (all of which were used in the ‘good' set and 30 of which were used in the ‘bad' set) and 13 media (all of which were used in both the ‘good' and the ‘bad' picks), for 80 growth assays total (actually 76, after removing contaminated samples—see Supplementary Data 5 for details).

**Figure 8:**
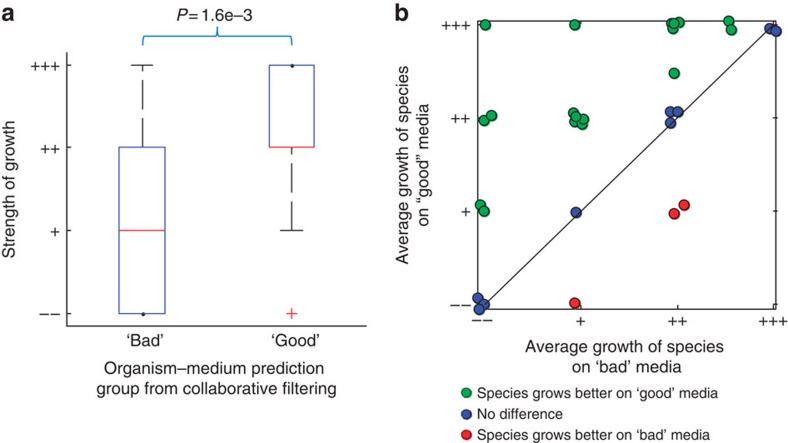
Growth of organisms on ‘good' versus ‘bad' media as predicted by GROWREC: A set of 40 ‘good' and 40 ‘bad' organism–media pairings were chosen by GROWREC, using the same 36 species and 13 media for both sets (but swapping which organisms paired with which media; 4 pairs were removed because of contamination). All organisms are aerobic heterotrophs with low salt requirements. (**a**) ‘Good' organism–media pairs showed significantly better growth than ‘bad' pairs (*P*=1.6e−3 in ranksum test). (**b**) Growth was also better on ‘good' versus ‘bad' media on an organism-by-organism basis (*P*=2.5e−3 in paired signrank test for each organism growing better on its ‘good' versus its ‘bad' media). Each circle in the plot represents a single organism, with its average growth on ‘good' media on the *y* axis, and its average growth on ‘bad' media on the *x* axis (dots are jittered for visibility). Organisms are coloured based on whether they grow better on their ‘good' versus their ‘bad' media (see legend).

This assay showed a striking difference in the strength of growth between the ‘good' and ‘bad' sets ( Fig. 8). The good group outgrew the bad group significantly as a whole (*P*=1.6e−3 in ranksum test based on quantized strength of growth assessments, as per Fig. 8a) and on an organism-specific basis (*P*=2.5e−3 in paired signrank test, as per Fig. 8b). This last finding indicates strongly the practical usefulness of GROWREC, as it enabled the choice of a better medium than a random selection might have yielded in over half of the organisms tested. It also reinforces that the relationship between organisms being close phylogenetically and their sharing lab media (that is, that shown in Fig. 3) is not merely descriptive, but can be used predictively to determine new organism–media pairs.

## Discussion

A large effort among microbiologists for over a century has been the development of suitable lab media for growing microorganisms. This has led to a broad range of artificial medium conditions of varying complexities that have been used to cultivate microbial life. Here, we map this culture media landscape by developing a database to incorporate the specific compositions of thousands of media, to link organisms with media and with the latter's respective components, and to link all of these elements out to other relevant growth-related features and/or databases. The resulting resource enables extensive integration of growth media information with existing tools such as genomic and metagenomic data and large-scale metabolic models, to facilitate predictive metabolic research. Such a resource has never before been produced at this scale. We make this resource publically available in the form of a web server that predicts media for bacteria and archaea given an input of 16S rRNA gene sequence, running on a GROWREC engine. We also provide in Supplementary Data 2 a large set of new organism–media predictions from our method, and in Supplementary Note 3 and associated Supplementary Figs 4–8, a range of analyses of how growth conditions differ among microbial phyla. Taken as a whole, this work represents a first ever large-scale analysis of the conditions implicit in growth media among the cultivated universe, as well as a platform for making new predictions of organism–media pairings within this space.

We see this work as a first step towards the goal of a more predictive science of microbial nutrition, in which information embedded in guides such as Bergey's Manual of Systematic Bacteriology can be mined for their insight and used alongside large and ever-growing repositories of metagenomic data and genome-scale modelling^17^,^20^ to rapidly develop cultivation media for any organism. Thus, part of our aim is to uncover principles for growing microorganisms that may be universal across life, and thus useful for culturing as-yet uncultivated organisms as well. To this end, we reveal that although most strain-specific media instructions relate to common salts, it is rarer metal ions and vitamins and cofactors that are the largest differentiators among defined growth conditions for strains within a given genus. This complements recent observations that many previously uncultivated microorganisms require specific cofactors or rare compounds in order to grow, and thus that emphasis on these types of trace nutrients in developing new media is warranted^1^,^19^. Importantly, the common approach of providing a standard trace elements mixture may not work for difficult to grow species, and a more nuanced or combinatorial approach might be necessary. For example, media for most strains of the nitrogen-fixing genus *Azotobacter* contain ∼20 μM molybdenum (which is used by the nitrogenase enzyme in these species), which is two orders of magnitude higher than the concentration in most molybdenum-containing media. The emerging importance of trace elements also raises the question of what other kinds of trace compounds might be important for developing culture media. Recent evidence shows that growth factors such as quorum sensing molecules and siderophores provided in bacterial co-cultures can enable growth of otherwise unculturable organisms^23^. These are important nutrients to explore in future culturing efforts.

We do not go so far as to cultivate uncultivated species in this study. However, we do show proof-of-principle that our GROWREC-based predictions can aid efforts to culture new organisms through two analyses: (i) by predicting media richness preferences of bacterial strains with high accuracy (Fig. 6) and (ii) by correctly predicting that xylan-consuming bacteria require polysaccharide nutrient sources, based solely on their 16S rDNA. Notably, using GROWREC in the manner we did for these analyses enables prediction of specific nutrients (here, media richness and polysaccharides), which can be done in principle for any nutrient deemed important, and for any organism regardless of whether it has previously been cultured. These analyses thus lay groundwork for future extensions that aim to design new media for as-yet-uncultured organisms, using observed and consistent principles that have been derived from the tree of cultivated life.

A major limitation of the work presented here is its reliance on a highly under-sampled organism-by-media growth matrix by which to train our predictors and derive insights. Namely, no organisms are listed as *not growing* on a given medium, and many organism–media pairings that could support growth are not listed in the DSMZ repositories (upon which KOMODO is based). This reflects the fact that researchers are usually interested in finding one or two good media for growing a particular strain, rather than in building consistent databases that can be mined for universal insights into microbial metabolism. The degree of accuracy we achieve in our new organism–media predictions despite these limitations attests to the enormous and largely yet-untapped potential of these approaches. Future work should focus on better populating subsets of the organism–media matrix deemed most critical for uncovering key growth principles, which can be done by performing many new growth experiments where a large set of organisms is tested on a large set of media in an all-grown-on-all fashion. The extent of such work will strongly dictate the depth of insight that can be derived and then aimed towards ‘unculturables'. In addition, many of the media that are in common use contain complex ingredients (for example, beef extract or peptone). In developing GROWREC, such undefined media are an asset, as they greatly expand our training set for making predictions. However, improvements should also focus on determining defined replacements for complex components, as these components obscure the signal of which nutrients are truly important for growing organisms in different phylogenetic groups.

## Methods

### Building KOMODO, the known media database

*Overview*. A large collection of media recipes for microbial strains is available through the German Leibniz Institute DSMZ strain and media collection (accessible here: https://www.dsmz.de/?id=441). These recipes are publicly available, but they are contained as instructions in PDF files that must be searched on an organism-by-organism basis. Putting these recipes into a usable database form required extensive and non-trivial work parsing, merging and organizing, as well as handling cross-references between media and submedia compound mixtures such as ‘trace element solutions,' which could be detailed and referenced from any DSMZ medium.

Dealing with such cross-references involves handling multiplication of volumes, masses and concentrations, even in cases when the same media component is included both in a submedium mixture and in the main medium description, often with non-matching names and/or units (for example, once in gram per litre and once in moles per litre). Medium and submedium volumes also are often not listed in media, but are assumed by microbiologists to be 1 l per the number of grams (or moles, or millilitres) of compounds listed for inclusion. However, there is no general rule for this, as some media do list specific volumes, some of which do not sum up to 1 l. Often, the volumes are left to be deciphered through common sense.

We tackled these challenges with a pipeline that is part manual and part automated. We used this pipeline to read in more than 1,500 PDF media descriptions and to create the KOMODO database, containing media compositions with standardized units. The pipeline is depicted as a 15-step process in Supplementary Fig. 1. Each step is explained in detail below:

*Steps 1–3: Manual standardization of media descriptions*. First, PDFs of all the media in the DSMZ database were copied verbatim into a text file. Next, the resulting ∼27,000 lines of text were manually reformatted in a way that could be machine read, using tags such as /ph/ (set the pH tag of the medium), /replace/ (replace one compound with another), /conc/ (change the concentration of a compound) and /rm/ (remove a compound from the medium) to denote media features and instructions. These tags were embedded in a specialized syntax that was similar to natural language media instructions, and thus required minimal alterations from the instructions listed in the original PDFs, but that followed a defined syntactical structure that could be interpreted by a computer programme. We were able to extract and reformat the majority of media from the DSMZ database in this way.

We noticed that a large number of organisms had specialized growth instructions listed either within the media descriptions or in the organism–medium mapping file provided to us by DSMZ. We considered these instructions critical to building an accurate database. To incorporate them, we copied the components of the base media and then implemented the stated changes to create medium definitions for each media variant. In all, this process resulted in nearly a doubling of the number of media in the database, from 1,946 to 3,672. In the DSMZ listing (http://www.dsmz.de/?id=441), each medium is referenced by an ID number. We generated unique new media IDs for these media variants by following the base media IDs with a period (.) or an underscore (_), and then a unique numerical or text string.

In addition, many media included in their compositions submedia, which were to be mixed independently and then combined. To ease the formation of the database, we treated each submedium as an independent medium with a new medium ID of 2,000 or above. This then allowed us to calculate cross-references between media and submedia using a standardized methodology.

*Steps 4–5: Manual standardization of compound names*. Media components as listed in the literature are highly redundant and degenerate. For example, the compound sodium sulfide is listed in the database in at least nine different ways (sodium sulfide, sodium sulphide, Na_2_S × 9 H_2_O, Na_2_S × 9H_2_O and so on). To convert the database to the most versatile form, we manually mapped compound names to ‘semi-unique names' as an intermediate layer, and then finally to ‘unique names' that contained only the precise metabolites contributed to a medium by a metabolite. For example, the ‘semi-unique' name mapped to all original forms of sodium sulfide (including hydrated forms) from media descriptions is ‘sodium sulfide', and the ‘unique name' is ‘SEED-cpd00239#cpd00971#,' which precisely depicts the two SEED compounds (cpd00239=sulfide ion and cpd00971=sodium ion).

We defined three classes of unique names:

1. SEED compounds, which are denoted with a ‘SEED-' tag and then up to three SEED metabolites contained within them (for example, ‘SEED-cpd00239#cpd00971#').
2. Complex components, which are denoted with a ‘rich-' tag (for example, ‘rich-peptone'). (Note, this ‘richness' is not to be confused with media richness; rather, it denotes complexity (media richness is treated differently in the work). In the main text, complex components are presented with a complex- tag instead of a rich- tag. The two are interchangeable, and both denote complexity, not media richness.)
3. Other compounds, which are chemically defined but are not in SEED. These are simply written out in full (for example, ‘1,4-Naphthaquinone').

*Steps 6–7: Determining media volumes and unit multipliers*. A rule of thumb in microbiology media recipes is that the quantities of compounds listed are those needed to produce 1 l of final medium. Because of this, media volumes are often omitted (and assumed to be 1 l) or are explicitly accounted for by mixing of media compounds with 1 l of water. However, there are many exceptions to this rule, such as media or submedia compositions that include some volume of water that is not 1 l or that contain very small volumes of liquid (from, for example, addition of some volume of ethanol), which should not be considered the ‘final volume' of the medium by any means. It was critical to determine the exact volume of media in order to properly convert compound units into concentrations (see Steps 9–11 for details).

To deal with this, we classed media and submedia into categories called ‘fill' and ‘scale.' The ‘fill' tag means that whatever volume a medium has should be ‘filled' to 1 l, that is, that the volume listed should simply be ignored; the ‘scale' tag means that the concentrations of compounds listed in a medium description should be scaled up with the listed volume until that volume comes out to 1 liter. Media were classed as ‘fill' and ‘scale' using general rules, which were overridden in ambiguous cases by manual curation (filling and scaling pseudocode is listed below). Finally, we adjusted final volumes of ‘fill' media and then determined a multiplier for each ‘scale' medium and submedium composition in order to convert compound units from moles to moles per litre (see Steps 9–11 for details).

*Step 8: Unpacking cross-media references*. Large proportions of DSMZ media contain cross-references either to other media or to complex submedia (∼60% and >25%, respectively). Many of these references also contain references, so sometimes multiple layers of references must be unpacked in order to build a given medium. Faithfully unpacking these cross-references requires (i) determining the molar concentrations of all compounds in the cross-referenced submedium/medium, (ii) determining the volume of the submedium/medium per litre of final medium, (iii) multiplying these two factors correctly to get the concentration of each submedium compound and (iv) accounting for the volume of the cross-referenced submedium/medium in determining the final medium volumes. This process was fully automated.

*Steps 9–11: Converting concentrations into moles per litre*. A goal of this project was to include every compound if possible with standardized units, as this would ease analyses between media and between compounds. Compounds in the original media files were listed with over 30 distinct units. As a first step, we built a mapping with multipliers to convert all of these units into five standard ones: gram per liter, liter per liter, moles per liter, trace and ‘gas substrate'.

The next step was to convert all of these units (except for the ‘trace' and ‘gas substrate' ones, which were treated separately) into Moles. To do this, we needed to obtain the molecular weights of all defined media components, as well as the molar ratios of each component forming each semi-unique compound name. When available, molecular weights of SEED compounds were taken from the SEED database. For SEED compounds without molecular weights listed, as well as for compounds falling into the ‘Other' category (that is, defined but not listed in SEED), we curated molecular weights manually based on Internet searches. Finally, we manually curated molar ratios of compounds in the original compound names, as well as the number of waters linked to each compound. With all of this information, we were able to calculate from, for example, the compound name ‘CoCl_2_ × 6 H_2_O,' the exact molar amounts of cobalt and chloride in a final medium composition, even if the original compound was listed in grams and not moles.

For the subset of compounds listed with units of volume rather than grams or moles, we universally assumed that the densities of the fluids were equal to the density of water (1 g ml^−1^), in order to ease the conversion of units. This rule was not used for volumes of submedia or media, but only for units of individual compounds.

Finally, we needed to convert the units for each compound from a molar amount (moles, M) into a molar concentration (moles per litre). This was done by multiplying the molar amount of each compound by the medium volume multipliers as determined in steps 6–7.

*Steps 12–14: Validating steps 6–11 versus gold standards*. Many complicated bookkeeping calculations are automated in steps 6–11 of this workflow, and there are many potential sources for mistakes or errors. Therefore, it was important to validate several key results as a sanity check in order to ensure that the database was faithfully converted. To do this, we manually produced three ‘gold-standard' files for validation:

- Manually calculated media volumes for 149 media and ‘fill' or ‘scale' statuses for 138 media, to check against the results of step 6.
- Manually calculated quantities (including units) of 973 compounds referenced across media, to validate the results of steps 7–9.
- Manually calculated molar concentrations of 965 SEED compounds in media, to validate the results of steps 10–11.

These files were used for extensive troubleshooting and debugging of the conversion code and of the syntax in the files for conversion, until there were no mismatches left between the manual files and the automated results.

*Step 15: Integrating media information into KOMODO*. The work in steps 1–14 ultimately produces a high confidence matrix of media versus the concentrations of compounds within them. This information was next integrated into a database format, along with the information provided by DSMZ of which organisms grow on which media, and linkages of DSMZ organism IDs to NCBI IDs and SEED organism IDs, when available.

*Pseudocode for automated portion of database build*. Here we provide pseudocode for steps 6–11 of the database building process, which are the automated portions for reading in the initial database information:

1. Determine volumes of each of the media.
2. Adjust volumes based on the following formula:
3. Determine the amount of each compound in each medium. For this, parenthesis are multiplied out (for example, (100 ml)*(5 g l^−1^) → 0.5 g l^−1^), with the general principle that all compounds are in units of mass or moles (that is, gram per litre or moles per litre). A compound that has a volume should be converted to grams using the formula: 1 ml=1 g (even though this is not strictly accurate, it is a reasonable approximation for most compounds we are dealing with). Also, submedia are treated like more embedded parentheses. For example, if medium a contains 10 ml of medium b, medium b contains 15 ml medium c and medium c contains 5 ml of metabolite X, then medium a contains (10 ml l^−1^)*(15 ml l^−1^)*(5 ml metabolite X)*(1 g ml^−1^ conversion)=0.00075, g metabolite X. Percentages are converted as shown in the conversion sheet.
4. For all SEED compounds, convert grams into moles. For this calculation, water molecules that are attached to the compound molecules should be accounted for. Water molecules that should be accounted for are always in the form ‘metabolite × N H_2_O'. For example, the metabolite: /notag/ CaCl_2_ × 2 H_2_O @ 10 mg would be converted as such:

*Coupling with SEED*. An ultimate goal of this work is to combine the knowledge embedded in manually built media with modern sequencing and genomics databases, in a form that may be used for large-scale metabolic analysis. A natural choice for this linkage is the Model SEED, a project that utilizes the RAST genome annotation server to automatically build and store genome-scale metabolic models^16^,^24^. To this end, we converted all compounds that had SEED equivalents into SEED compound names and IDs, with each ingredient listed in a medium converted into between one and three SEED compounds (see example in Fig. 1). The quantities of these SEED compounds (as well as compounds without SEED equivalents) were then combined in final media compositions and converted to molar units.

*Future work on KOMODO*. Future work that can be done to improve KOMODO is described in Supplementary Note 1.

### Choosing organism–media pairs for experiments

We describe three sets of new *in vitro* experiments in this study: (i) validating transitive predictions; (ii) validating collaborative filtering predictions and (iii) determining whether highly ranked collaborative filtering predictions grow better than low-ranked predictions. As we had many predicted organism–media pairs to choose from for running each set of experiments, we chose pairs to test based on the following criteria:

*Experiments 1 and 2: Transitive and collaborative filtering*. For these experiments we chose organism–media pairs that were: (i) the highest ranked based on our predictions (hence ‘top'); (ii) convenient for our collaborators at the DSMZ to test (mainly, which involved media that are not difficult to produce and strains that are both under the care of the curators involved in the study and that are not known to be extremely difficult to grow) and (iii) contained some organism–media pairs that the curators deemed likely to grow, and some that they were unsure or negative about. Transitive pairs we validated were also chosen based on curator preferences for working with certain organisms and media.

As just mentioned, we tried to choose an assortment of pairings that curators had guessed would yield growth, and also of pairings that curators gave a ‘maybe', ‘no' or did not have an idea about. Of transitive predictions tested, 24 were predicted beforehand by the curators to yield growth and 19 were either indeterminate or were predicted to not yield growth. Of collaborative filtering predictions tested (in the original experiments), 38 were predicted beforehand by the curators to yield growth and 23 were indeterminate or no.

*Experiment 3: Good versus bad predictions*. For this experiment, we chose organism–medium pairings by optimizing for four factors (with decreasing levels of strictness): (i) using only a set of organisms and media that our experimental collaborators at the DSMZ told us they preferred to work with; (ii) maximizing the GROWREC scores of the ‘good' group as selected from within these organisms and media; (iii) minimizing the GROWREC scores of the ‘bad' group and (iv) maximizing the number of organisms from the ‘good' group that were also used in the ‘bad' group. All media were used in both, but it was not possible to design experiments where every organism would be included in both the ‘good' and the ‘bad' group while still maintaining a large difference in the collab scores, given the set of organisms and media we could work with; hence, we maximized the number of organisms shared between the groups given the other constraints. No organisms were included in the ‘bad' group that were not included in the ‘good' group, but a few were in the ‘good' group and not the ‘bad'. The collaborative filtering scores for the ‘bad' group were determined after relaxing the phylogenetic distance cutoff built into the GROWREC predictor, thus enabling it to be highly permissive and to determine scores for phylogenetically distant organisms. These scores were in general very low; we selected the lowest for the ‘bad' group, as just described.

### Calculating partial media matches

To compare phylogenetic or ecological similarity to partial matches between the known lab media that two organisms can grow on, we considered the (i) union or (ii) best matching of media each organism grows on in KOMODO. Within each ecological/phylogenetic distance bin, we took the fraction of organism pairs whose media were similar above some (iii) Jaccard threshold or (iv) count of components. All combinations of these four methods were tried (see Supplementary Fig. 13a–d for details). For analyses of the counts, it was important to examine thresholds that were lower than the number of components in any of the examined media (otherwise, even two organisms that exactly share a lab medium would not be considered to ‘match'). Therefore, for this analysis, we examined only media of 15 or more components, and we only considered thresholds up to 15 (see Supplementary Fig. 13b,d for details).

### Determining ecological or phylogenetic similarity

Ecological similarity between pairs of organisms was determined using a Jaccard metric of co-growth in the Greengenes database; that is, the number of environments that both organisms grow in, divided by the total number of environments that either organism grows in (this was done in the same manner as in ref. 17). Phylogenetic similarity was determined as (1−normalized phylogenetic distance), where phylogenetic distance is the number of organisms from the NCBI taxonomic tree beneath the lowest common ancestor of two organisms (that is, subtree distance) divided by the total number organisms in the tree. Pairs of organisms were then binned by ecological (or phylogenetic) distances, and the fraction of pairs in each bin sharing a lab medium according to KOMODO was used as the final ‘medium distance' for that ecological distance bin (results plotted and Spearman correlations given in Fig. 3).

### Testing transitivity of organism-media pairings

First, we excluded media that have <3 or >100 organisms listed as growing on them. We define an XY|_m_ event as organisms X and Y each being listed in the KOMODO database for medium m. We then identified 1,000 cases in which AB|_m1_, BC|_m2_ and C|_m3_, enforcing that media m1, m2 and m3 are different and that organisms A, B and C are different. Given the relations stated above, for each of the 1,000 cases we checked the number of instances of CA|_m3_ (test case) versus the number of instances of CX|_m3_ (random control), where X was a randomly chosen organism from the database. Test cases that were positive were counted as true transitive events and random control cases were considered to be an estimate of noise.

### Building GROWREC

To predict new potentially viable organism–media pairings, we developed a collaborative filtering predictor as follows: first, phylogenetic or ecological distances between pairs of organisms are determined. Phylogenetic distance is determined by the count of organisms beneath the lowest common ancestor in the ITOL taxonomic tree (subtree elements count), and ecological distance is determined via a Jaccard metric of co-growth across environments listed in Greengenes^25^, as clustered previously by Chaffron^26^. Unless otherwise specified, we used phylogenetic distance for analyses.

To determine a collaborative score for the likelihood of a ‘test' organism growing on a given medium, we count how many organisms phylogenetically related to the test organism can grow on it. We chose a distance cutoff and a weighting based on phylogenetic distance (cutoff was set at a normalized phylogenetic subtree distance of 0.04; weighting of the collaborative score was taken as cutoff/subtree distance; see Supplementary Note 6). The threshold for ecological distance was a Jaccard distance of 0.15, with no distance weighting. These cutoffs were chosen to bolster precision, using an analysis similar to those shown in Supplementary Figs 10 and 11.

Collaborative filtering predictions were validated using a leave-one-out method, in which media predictions made for organisms present in KOMODO were compared against true pairings of those organisms with lab media from the database. To predict media for a given organism, the organism was left out from the database, and its phylogenetic relationships to other organisms in the database were used as input to the collaborative filtering predictor.

As a validation of the collaborative filtering predictor, we developed another ‘null' predictor that is based solely on media and organism popularity in the database. This predictor pairs organisms with media randomly, with distributions of both organisms and media weighted by their commonality in known pairings in the DSMZ database. When predicting results to compare against the collaborative filtering predictor (which are the results presented in the present paper), we only considered organisms that are present in the phylogenetic data set.

### Improving GROWREC with O_2_ and salt filters

Filtering of collaborative filtering results based on biological features proceeded in two steps:

- We determined the preference of an organism for that feature by looking at instances in the known DSMZ organism–media database of the organism being paired with media containing that feature. If, for example, an organism always grows on a medium containing O_2_, then we label the organism as ‘aerobic'; if an organism always grows on an anaerobic medium, we label the organism as ‘anaerobic'; if it sometimes grows with and sometimes without O_2_, we label it ‘facultative'. For salt tolerance, a ‘salty' medium was one with ⩾15 g NaCl per l, or containing the word ‘sea'.
- Collaborative filtering results were then filtered based on mismatches between the preference of the organism and the contents of the predicted medium. For example, an ‘anaerobic' organism growing on an ‘aerobic' medium would be excluded by the filter, as would an ‘aerobic' organism growing on an ‘anaerobic' medium.

### Determining rich medium preferences

Media containing any complex component (that is, 1 of the 11 categories shown in Fig. 1) were broken into ‘low', ‘medium' and ‘high' richness groups based on a weighted sum of complex components present in the medium, using the weights in Supplementary Table 1. Cutoffs for the three richness groups were 5 g per l and 15 g per l of ‘rich' component (as determined through the weighted sum). The amounts of certain defined components were also included in the richness determination—these components are listed in Supplementary Data 1.

To determine the category that an organism falls into, we summed the collaborative scores of all low, medium and high richness media that the organism was predicted to grow on via GROWREC, after filtering results for oxygen and salt (that is, eliminating pairing of a high-salt medium with a low-salt organism and so on). The category with the highest combined score is the category chosen as the ‘preference' of the organism.

Predicted organism richness preferences were compared with manually curated preferences (from DSMZ curators) as well as a ‘gold-standard' set of richness preferences from KOMODO. These are known organism–medium pairings where the organism is only found growing on media within a given (predicted) richness category—low, medium or high. If an organism, for example, is known to grow only on two ‘low' richness media, then the gold-standard richness preference for that organism is ‘low'. The results of these comparisons are described in detail in Supplementary Data 3.

### Integrating GROWREC into an online platform

The GROWREC engine was used to produce an online tool that predicts media from the DSMZ repository most likely to enable growth of any organism. Users can input organisms in the form of a 16S rDNA sequence or an NCBI taxon ID. If a DNA sequence is inputted, the algorithm will use BLAST to determine related organisms, and then will perform collaborative filtering (in the manner of GROWREC) to predict viable media. Predicted media are given with their collab scores, which denote the strength of the prediction. The tool can be found at: http://delta-tomcat-vm.cs.tau.ac.il:40678/komodo/default.htm.

### Experimental methods

To test a given organism–medium pair, strains were reactivated from freeze-dried ampoules as recommended in the DSMZ catalogue (http://www.dsmz.de/catalogues/catalogue-microorganisms.html) and incubated on the medium used as standard for this organism. After incubation at the temperature recommended for the specific strains by the DSMZ, colony counts and size were evaluated and subcultures were prepared in the predicted medium. Growth was documented after 24 and 48 h of incubation. Strong and medium growth were both considered positive growth phenotypes.

The strains used for experiments, and all of the results, are listed in the following documents:

Supplementary Data 5—Transitive experiments.

Supplementary Data 6—First set of collaborative filtering experiments.

Supplementary Data 7—Good–bad collaborative filtering experiments.

## Additional information

**How to cite this article:** Oberhardt, M. A. *et al*. Harnessing the landscape of microbial culture media to predict new organism–media pairings. *Nat. Commun.* 6:8493 doi: 10.1038/ncomms9493 (2015).

## Supplementary Material

Supplementary Information Supplementary Figures 1-15, Supplementary Table 1, Supplementary Notes 1-8 and Supplementary References

Supplementary Data 1 Transitive predictions.

Supplementary Data 2 Collaborative Filtering organism-media pairing predictions (phylogenetic-based predictor).

Supplementary Data 3 Organism richness preferences.

Supplementary Data 4 SEED compounds.

Supplementary Data 5 Transitive experiments (experiment 1). These are in vitro experiments done to validate predicted organism-medium pairings based on the transitivity property.

Supplementary Data 6 Collaborative filtering experiments (experiment 2).

Supplementary Data 7 Good versus Bad growth experiments (experiment 3).
