# Supplementary material for: Harnessing the landscape of microbial culture media to predict new organism–media pairings
Source: Nat Commun. 2015 Oct 13;6:8493. doi: 10.1038/ncomms9493 (PMC4633754; doi:10.1038/ncomms9493)
Supplement: Supplementary Information — Supplementary Figures 1-15, Supplementary Table 1, Supplementary Notes 1-8 and Supplementary References [file ncomms9493-s1.pdf]

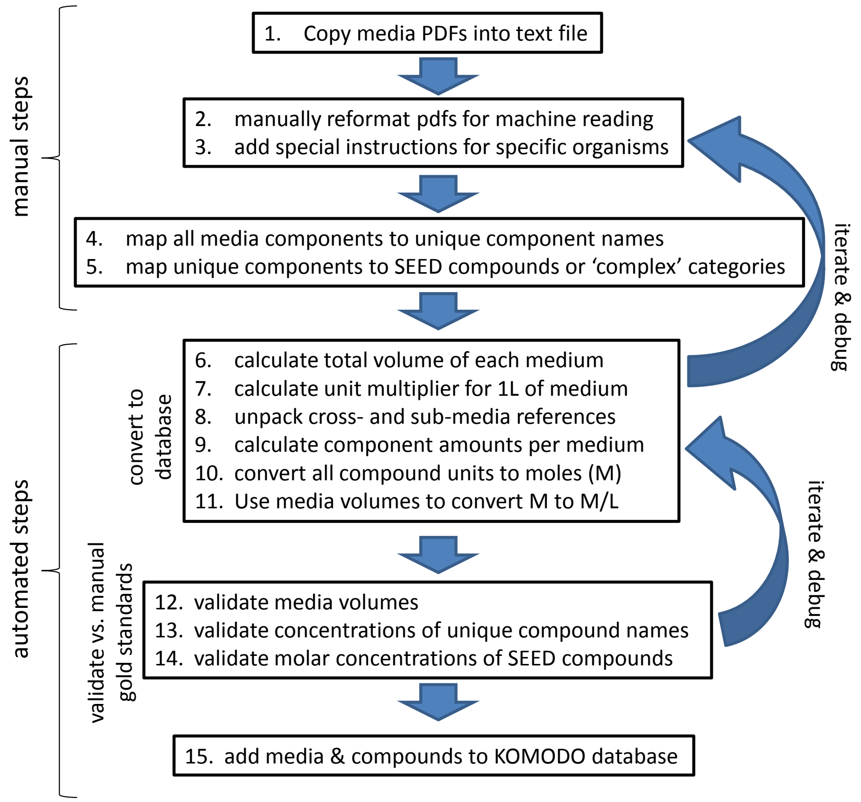

Supplementary Fig. 1. Workflow for building KOMODO, the Known Media Database. This partially manual and partially automated workflow enabled the building of KOMODO, based on media recipes publically available on the Leibniz DSMZ website. Individual steps of the workflow are described in detail in the Methods.

a.

% contribution of top compounds to PCs

compound:	pc1	pc2	pc3
Cl-	47	29	-3
Na+	48	-28	4
S	0	0	53
HCO3	1	-23	-12
H2S	0	-4	14
Mg2	1	5	-2
	**(94.6%)**	**(2.7%)**	**(0.5%)**

Information content of PC

b.

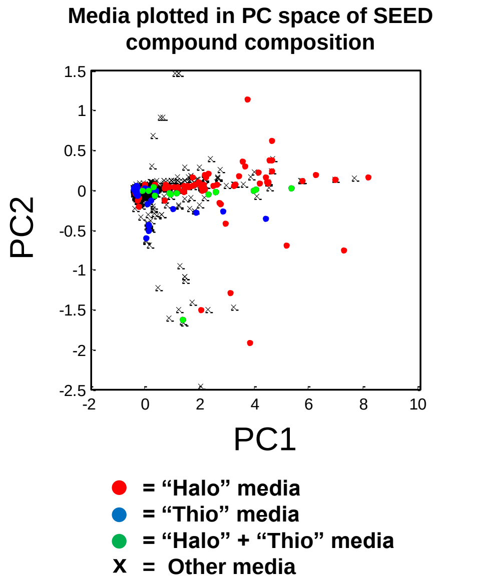

c.

After removing Na+, Cl-, and Pi

% contribution to media pcs:

	pc1	pc2	pc3
S	-2	76	-12
H2S	1	13	71
H2CO3	80	2	-1
Mg	-1	2	8
D-Glucose	5	-1	0
H2S2O3	5	0	1
	**(30%)**	**(17%)**	**(11%)**

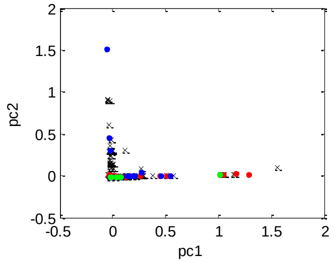

d.

All concentrations set to "1".

% contribution to media pcs:

	pc1	pc2	pc3
Zn2+	3	2	-3
H2CO3	2	-1	6
Sodium bicarbonate	2	-1	6
Cu2+	3	2	-3
Co2+	3	2	-3
Mg	3	3	2
	**(38%)**	**(10%)**	**(5%)**

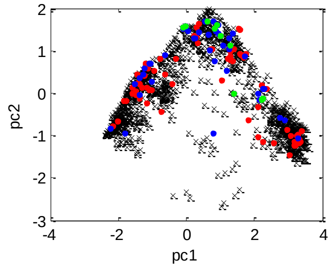

Supplementary Fig. 2. Principle component analysis of media compositions. A principle component analysis across a media-by-component matrix (with complex components excluded) reveals the importance of NaCl (salt) in differentiating media. (a) Compounds that dominate the first three principle components, normalized to the percent of the absolute value of all values in each PC (column). NaCl is dominant in PC1; Cl^- but not Na^+ is dominant in PC2; sulfur is dominant in PC3. The contributions of these PCs to the total information content is shown below. (b) All media are plotted in the PC1 / PC2 projection. Media containing the word 'Halo' (i.e., salty) or 'Thio' (i.e., sulfurous) in their names are colored red and blue, respectively; green are media containing both terms. Media with high PC1 coordinates are enriched for 'Halo' media, as expected. (c) The same plots are shown when Na+, Cl-, and Phosphate (Pi) are removed from the matrix before PCA. (d) The same plots are shown for PCA on the media-component matrix after converting all concentration values to "1" (if the component is present) or "0" if it is not. Colors in the PC plots are as in (b).

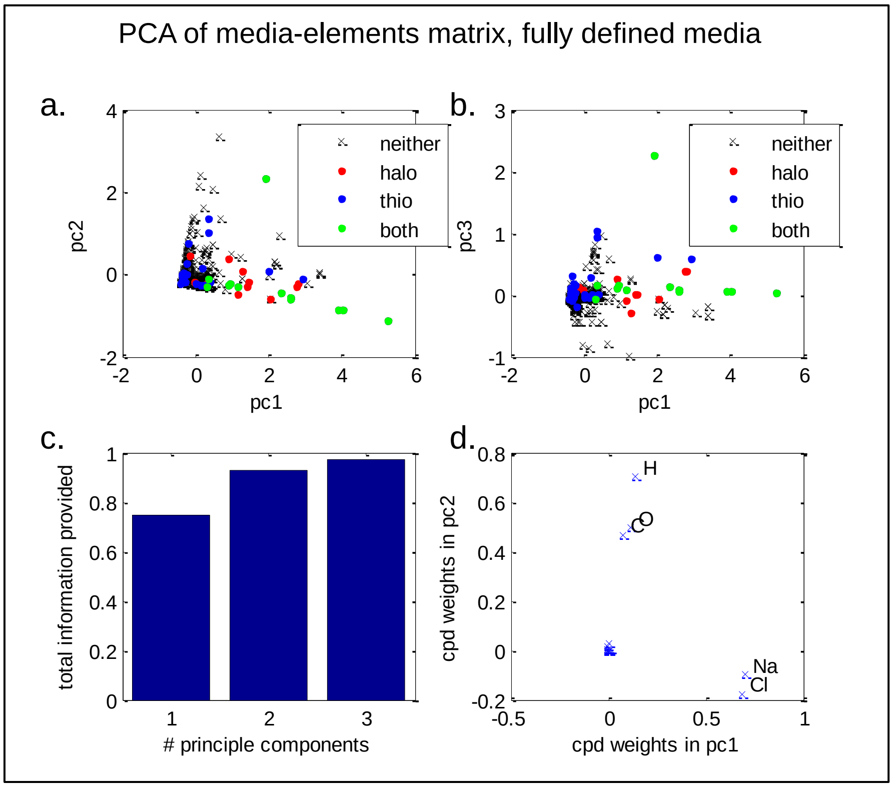

Supplementary Fig. 3. PCA of media by chemical element composition, fully defined media. This is a principle component analysis akin to that shown in Supplementary Fig. 2, but after media are broken down into elemental constituents. For example, 1 mol/l of SEED compound Glucose would contribute 6 moles Carbon, 12 moles Hydrogen, and 6 Moles Oxygen to a given medium in this matrix. All variants of medium 861 (SRB-PSYCHROPHILE MEDIUM) were excluded from this analysis because they have much higher concentrations of carbon compound than other media. (a,b) Media laid out on pc plots. (c) Informational contribution of the first 3 principle components to the total matrix. (d) Weights of chemical elements in principle components 1 and 2.

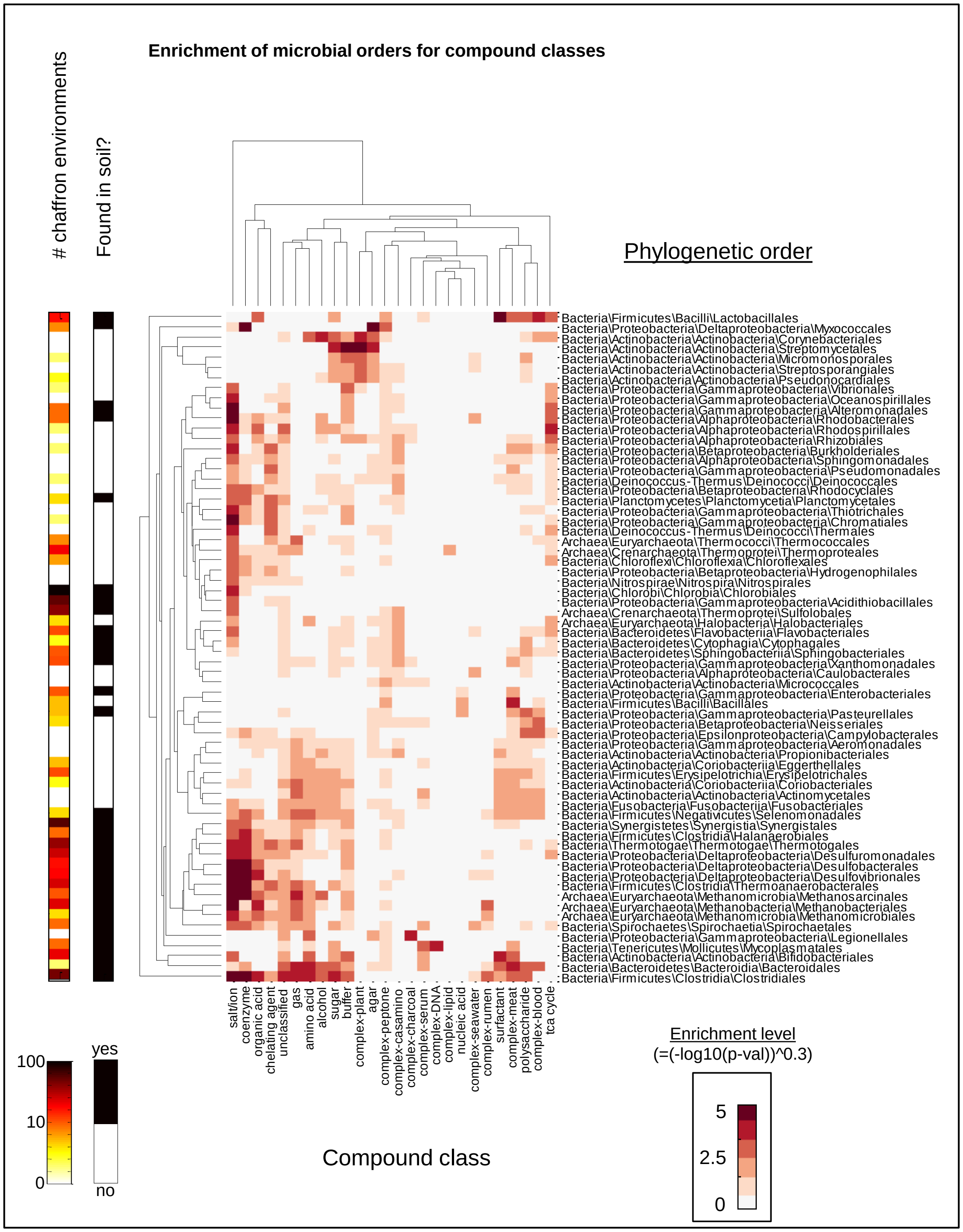

Supplementary Fig. 4. Clustergram of taxonomic Order vs. component category enrichment matrix. A clustergram of the enrichment matrix of Taxonomic Orders versus component categories. Bars to the left show whether any organisms from within the order are listed as soil bacteria (from our ecological dataset), and how many ecological environments the given organisms are listed as growing in (this relates to the Chaffron/greengenes environments, which are assignments of strains to ecological environments; see Methods). Note: the components belonging to all compound classes are listed in Supplementary Table 4.

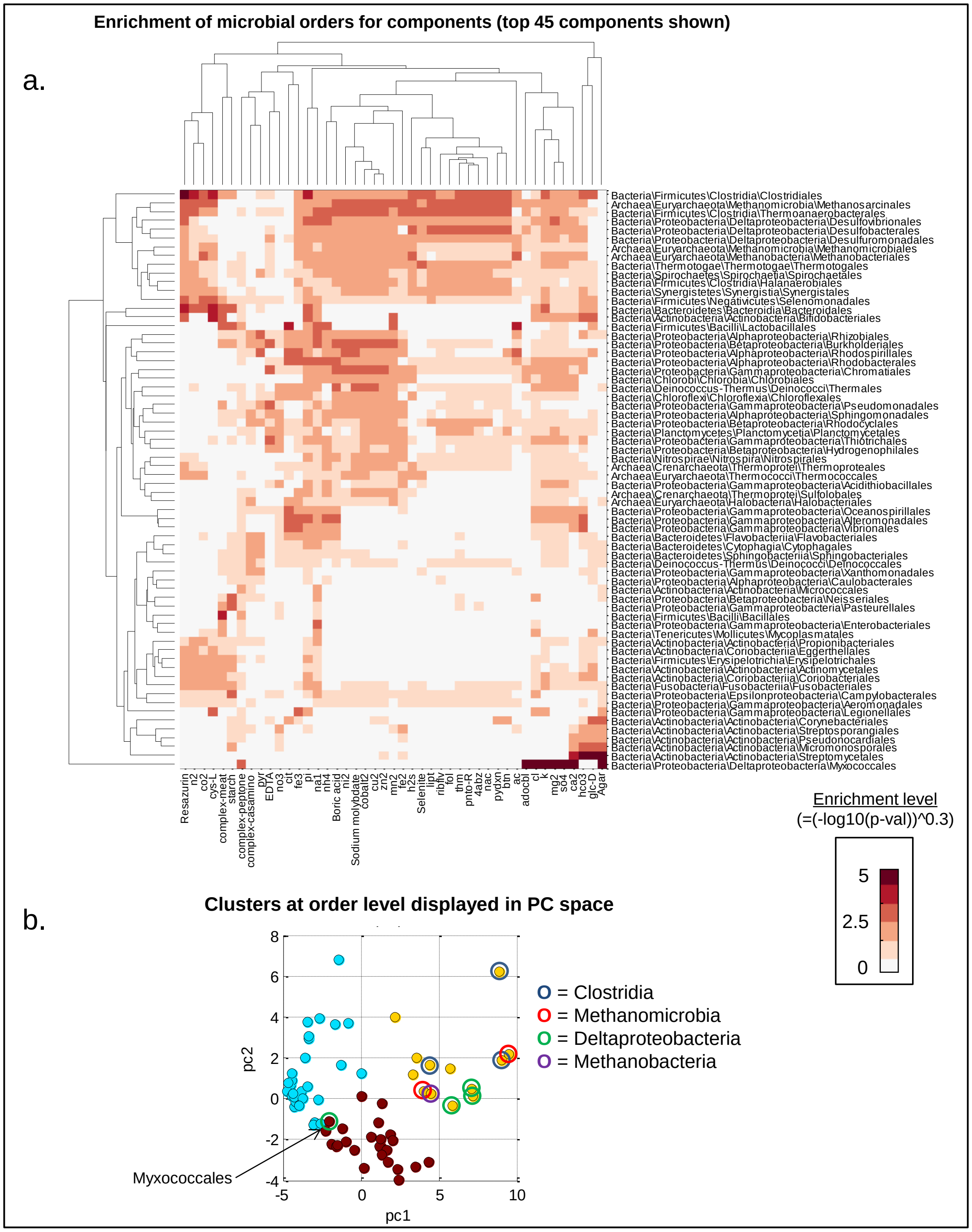

Supplementary Fig. 5. Clustergram of taxonomic Order vs. component enrichment matrix. An enrichment matrix of Orders versus individual media components was generated. (a) A clustergram of this enrichment matrix, with only the 45 most prevalent components shown (both X- and Y- axes are clustered). (b) The Orders are plotted in PC space from a PCA of the matrix in (a), and clustered into 3 groups. Taxonomic Orders belonging to Classes Clostridia, Deltaproteobacteria, and Methanomicrobia (as well as the one Order of the Class Methanobacteria) cluster together. Note: as in the other figures and as noted in the main text, the 'complex-peptone' component also contains yeast extract (hence, e.g., why Myxococcales, most of which grow on medium containing baker's yeast, are enriched for the complex-peptone category). Note: all compound abbreviations are elucidated in Supplementary Table 4, and shown here: Pyr = pyruvate; EDTA = Ethylenediaminetetraacetic acid; cit = citrate; pi =

phosphate ion; na1 = Sodium ion; lipt = lipoate; ribflv = riboflavin; fol = folate; thm = thiamin; pnto-R = pantothenic acid; 4abz = 4-aminobenzoate; nac = niacin; pydxn = pyridoxol; btn = biotin; ac = acetate; adocbl = coenzyme B12.

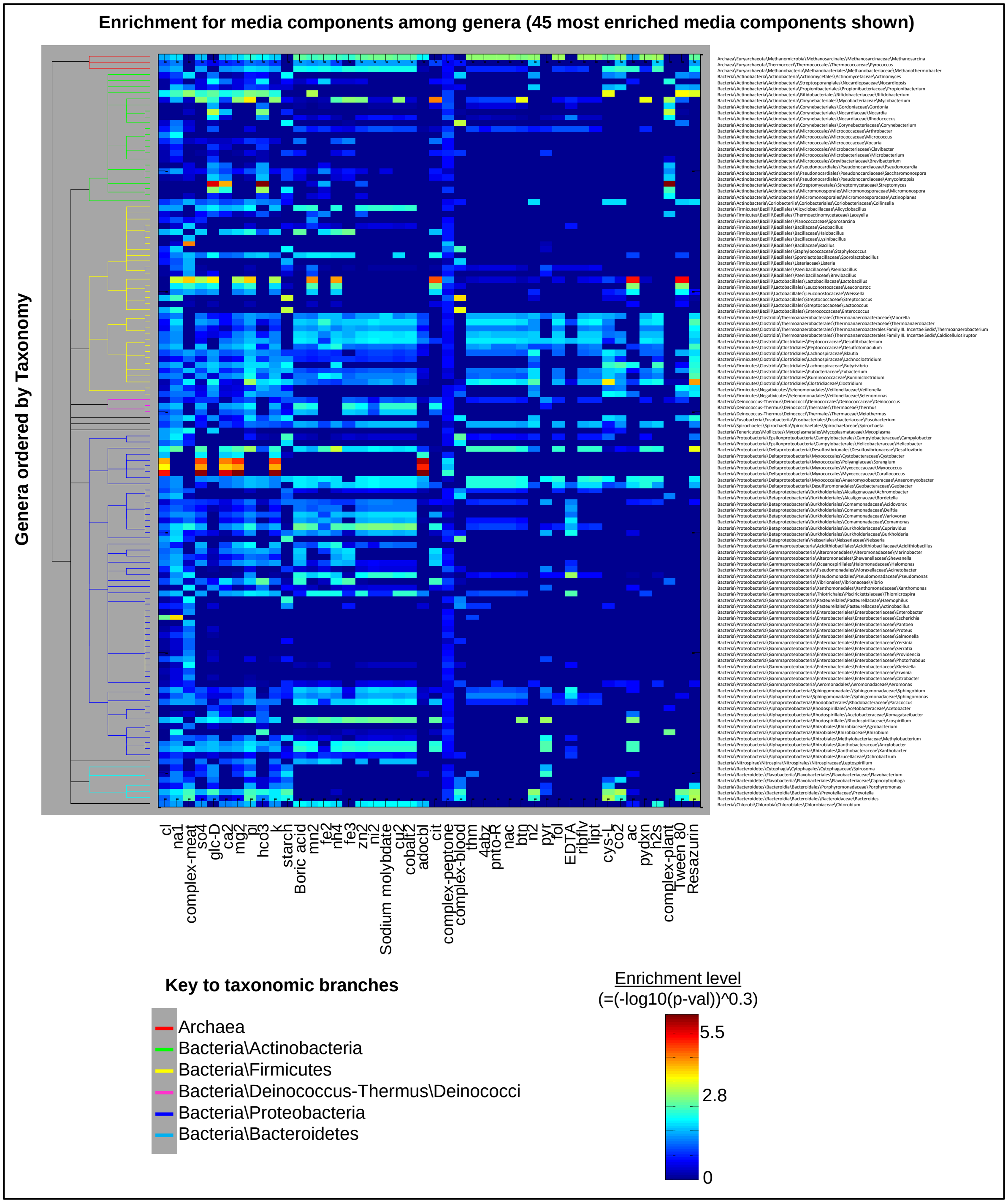

Supplementary Fig. 6. Enrichment matrix of Genera vs media components. The Y-axis is ordered by Taxonomy. The X-axis is ordered by prevalence (most to least). Only the most prevalent 45 components are displayed, and only Genera with at least 7 members listed in DSMZ are shown. Compounds abbreviations are as in Supplementary Figure 5.

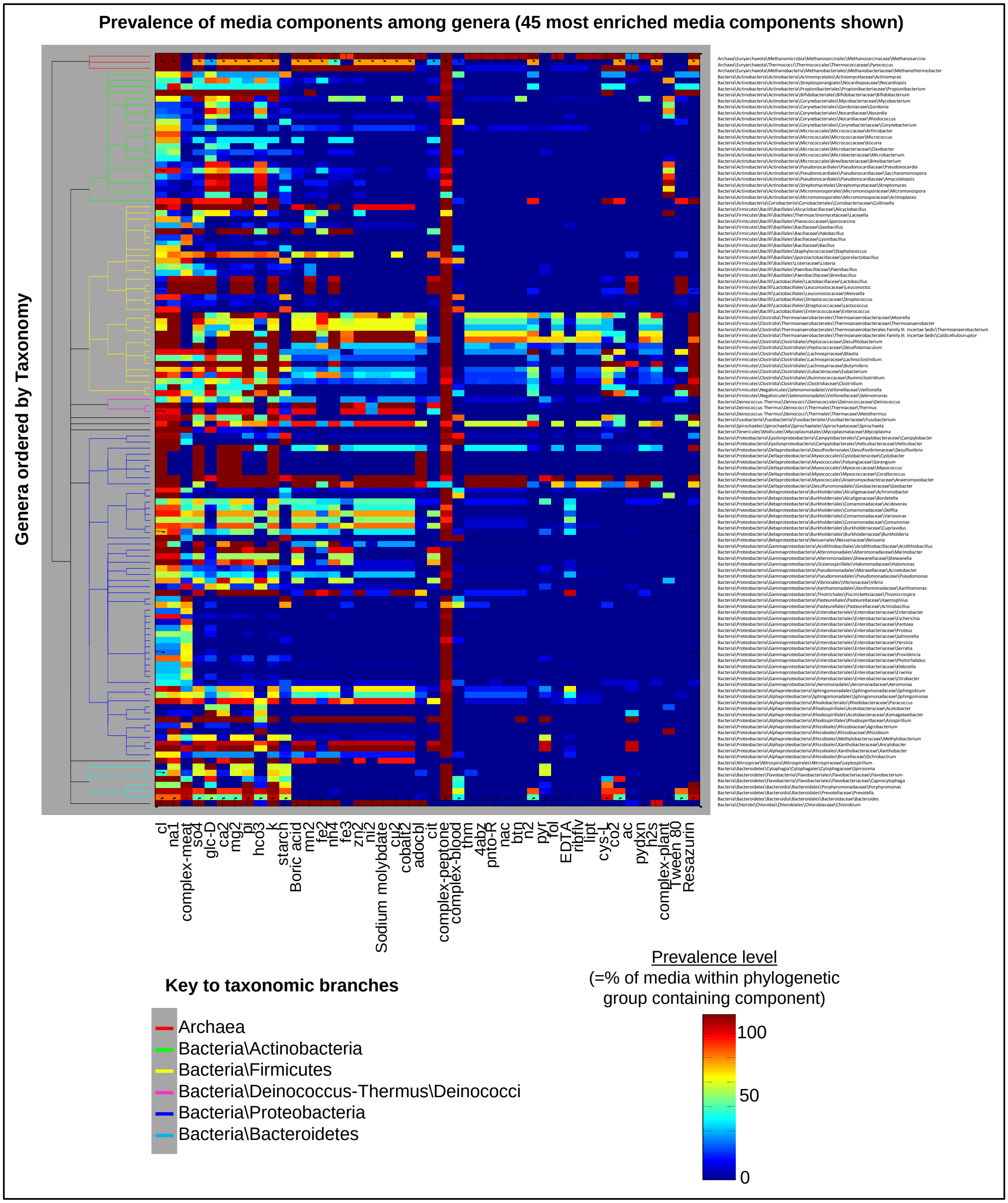

Supplementary Fig. 7. Prevalence matrix of Genera vs media components. The Y-axis is ordered by Taxonomy, and the X-axis is ordered the same way as in Supplementary Fig. 6, with the same compounds listed. As in Supplementary Fig. 6, only Genera with at least 7 members listed in DSMZ are shown. This matrix shows the prevalence of each component within the media for each genus, measured as the % of media in the genus that contain the component. This differs from enrichment as, for example, the complex-peptone/yeast category is prevalent across nearly all general, so it is not enriched in any (so it shows up in this matrix, but not in Supplementary Fig. 6). Compound abbreviations are as in Supplementary Fig. 5.

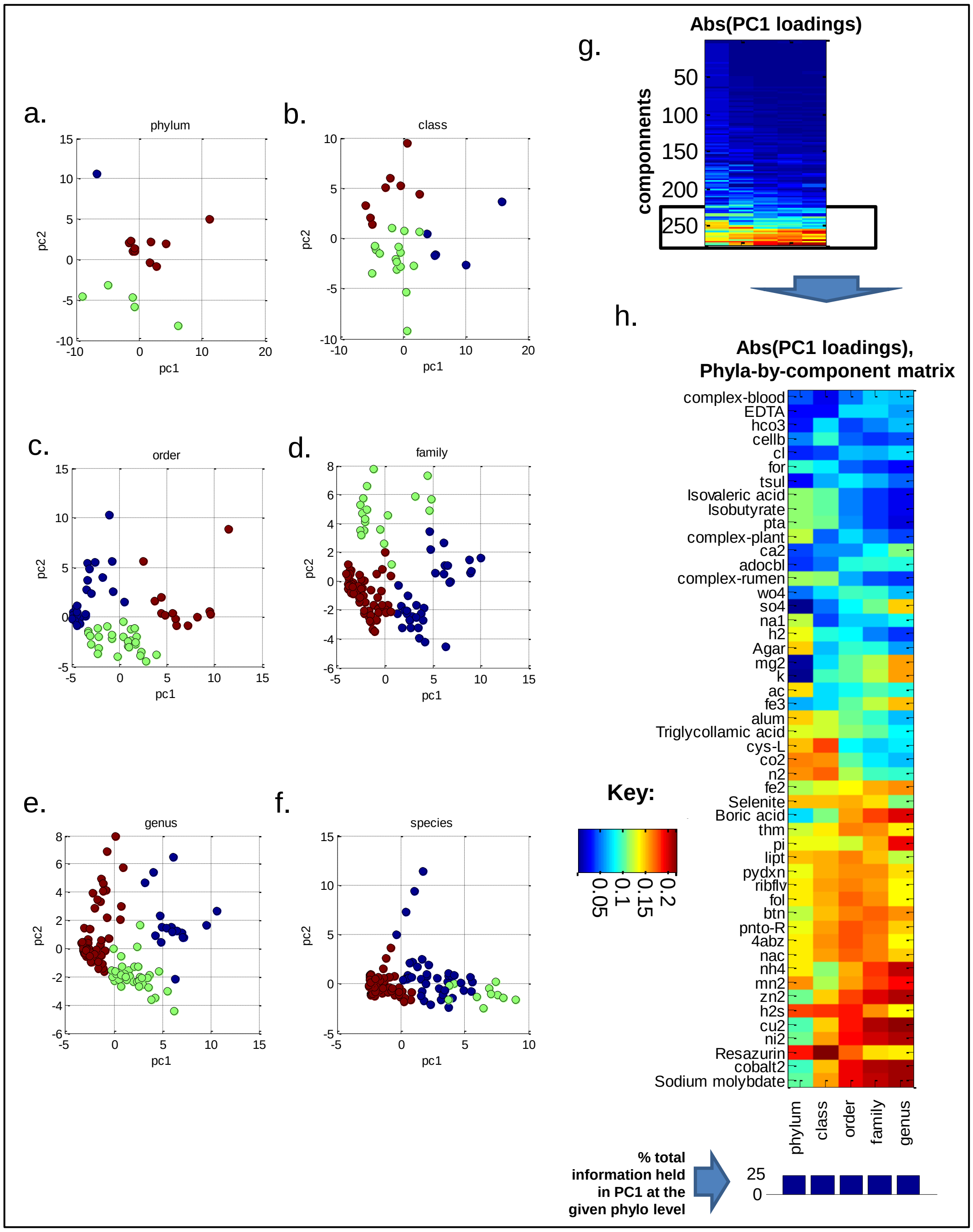

Supplementary Fig. 8. PCA of phylogenetic groups based on enrichment for media components. (a-f) Phylogenetic groups at different levels are grouped into 3 clusters. These clusters were calculated using the phylogeny-versus-compound matrix, using unsupervised K-means algorithm, with K=3 (i.e., phylogenetic groups are clustered together if the media components they are enriched for are similar; they are not clustered based on, e.g., phylogenetic similarity). They are then plotted on a PCA plot, which is angled to show maximal separation between clusters. (g-h) The loadings (i.e., weights) of compounds in the first principle component at each level is shown in the heatmap. (h) contains the bottom, highly weighted portion of the heatmap blown up. At each phylogentic level, phyla are only kept for analysis if they contain at least 7 members (within the DSMZ/KOMODO dataset).

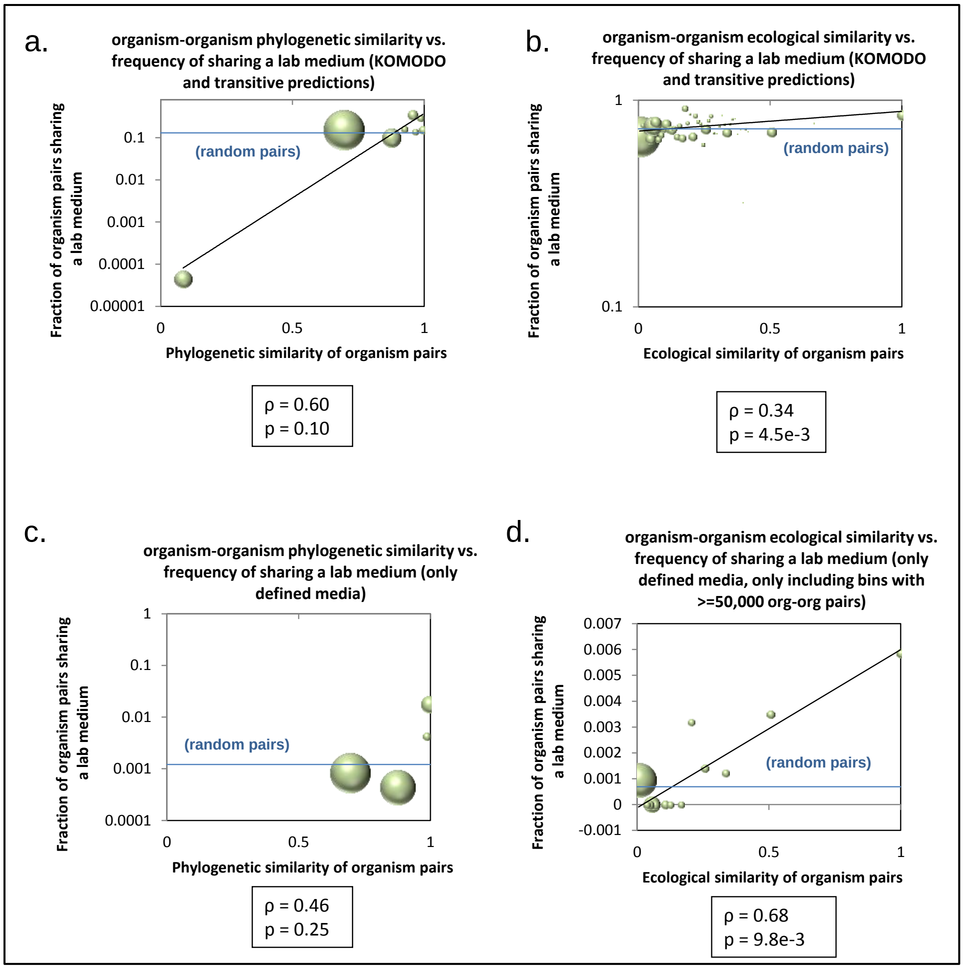

Supplementary Fig. 9. Chance to share a lab medium vs. phylogeny or ecological distance, variants. Following on Figure 3 in the main text, the chance for two organisms of a given phylogenetic (a & c) or ecological (b & d) distance to share a lab medium is analyzed for KOMODO augmented with the first round of transitive predictions (a-b) or for only the defined media in KOMODO, with complex media excluded (c-d). Correlation values are Spearman correlations.

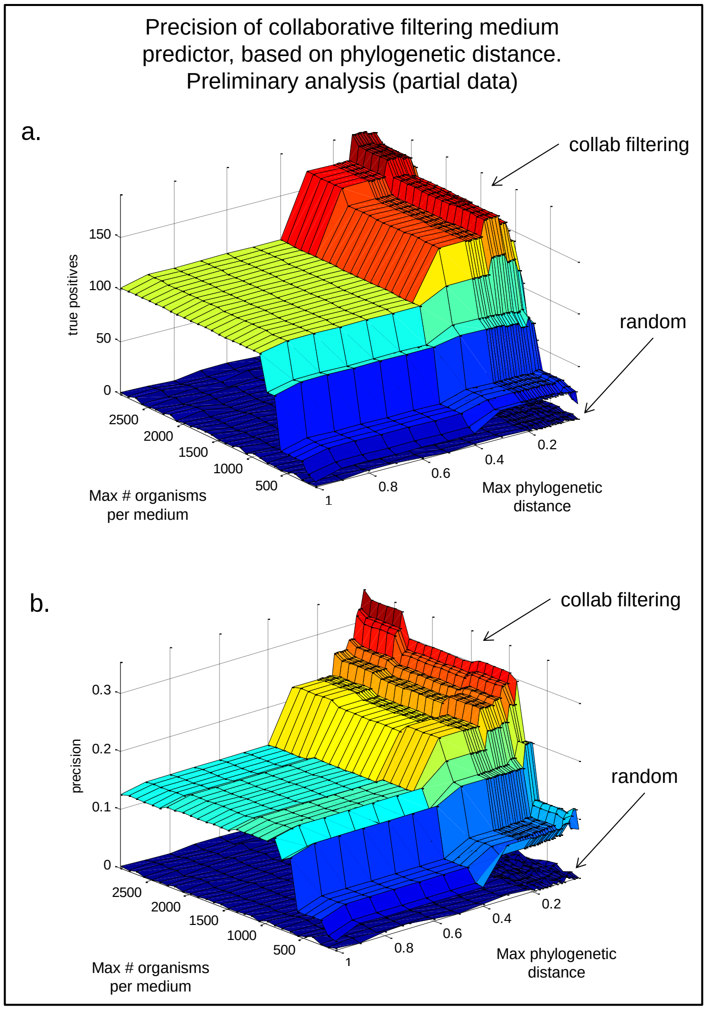

Supplementary Fig. 10. Precision of collab filtering predictor vs. random picks based on media frequency. A demonstrative example of the improved results of the raw collab filtering results, before a phylogenetic distance cutoff was chosen.

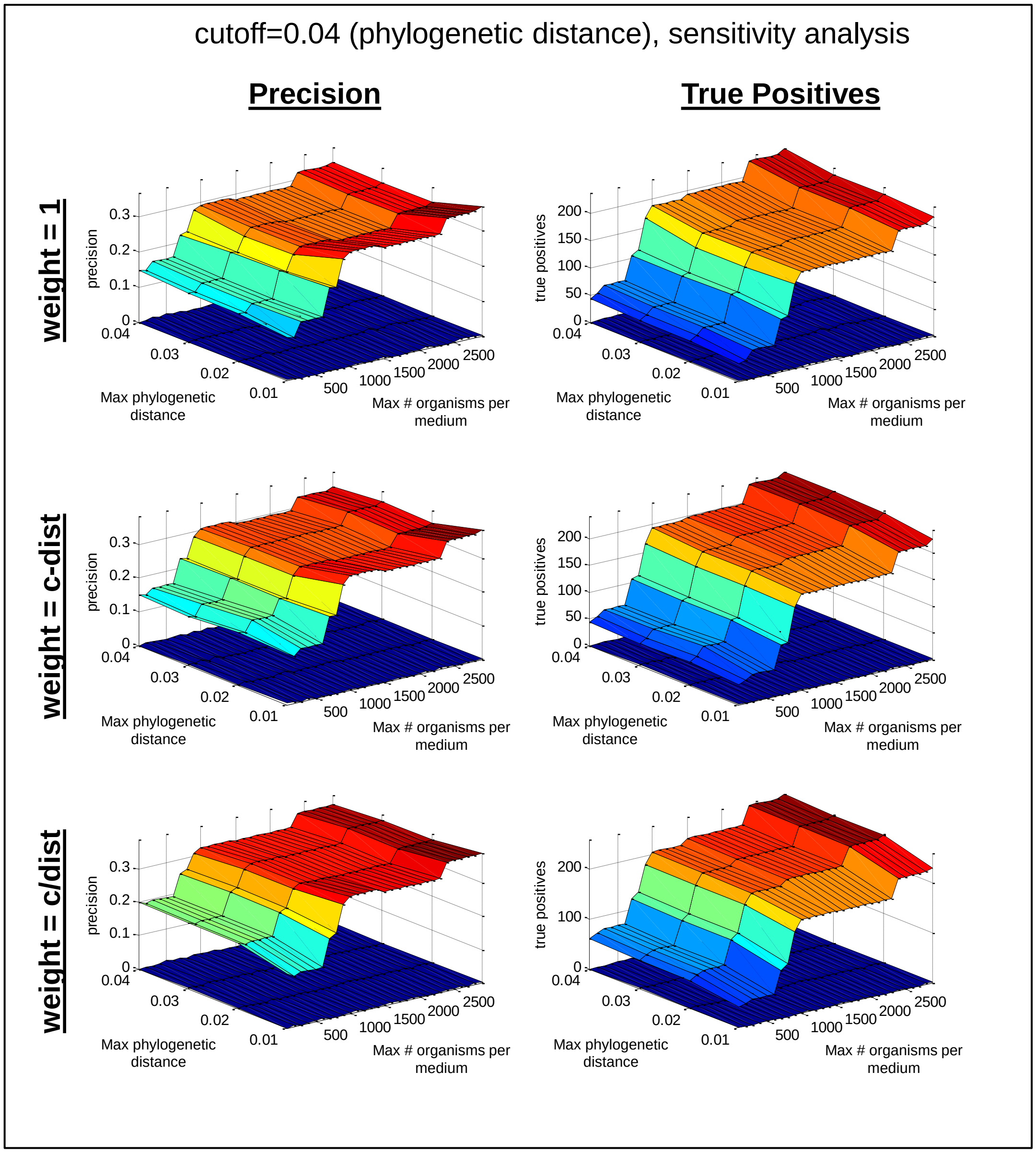

Supplementary Fig. 11. Different weightings on phylogenetic distances in collab filtering output. Demonstrative output of an analysis of precision and true positive percentages gained when we used different weightings and cutoffs for determining collab score in GROWREC. The final weighting (c/dist, where c=0.04 phylo distance) was chosen for subsequent analyses.

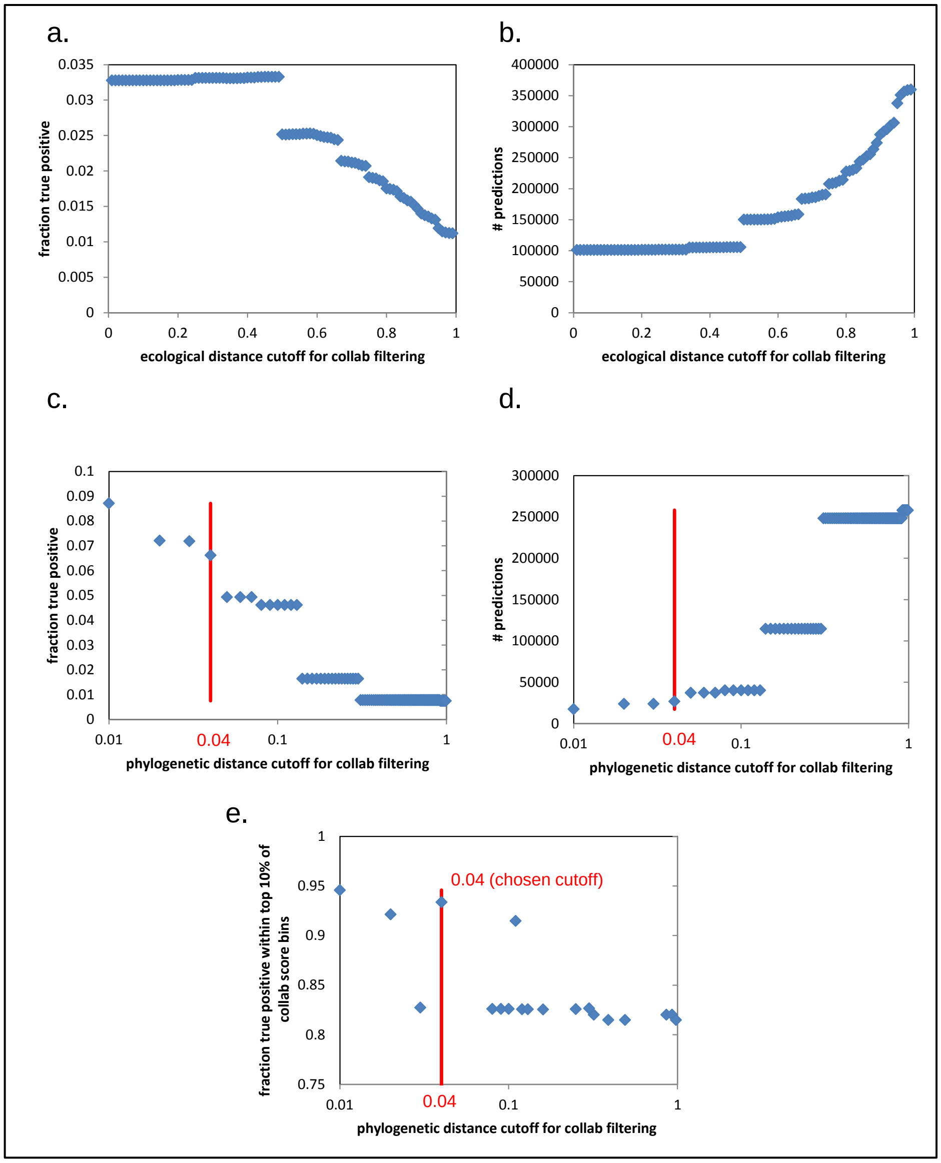

Supplementary Fig. 12. Effects of phylogenetic or ecological distance cutoff on accuracy of collaborative filtering predictor. Fractions of true positive results (subplots a and c) and the total number of organism-media pairs predicted (subplots b & d) are shown for ecological-based and phylogenetic-based collaborative filtering predictors, using different cutoffs (x-axis). (e) the fraction of true positives in the top 10% of collab scores (after binning) using a phylogenetic collaborative filtering predictor.

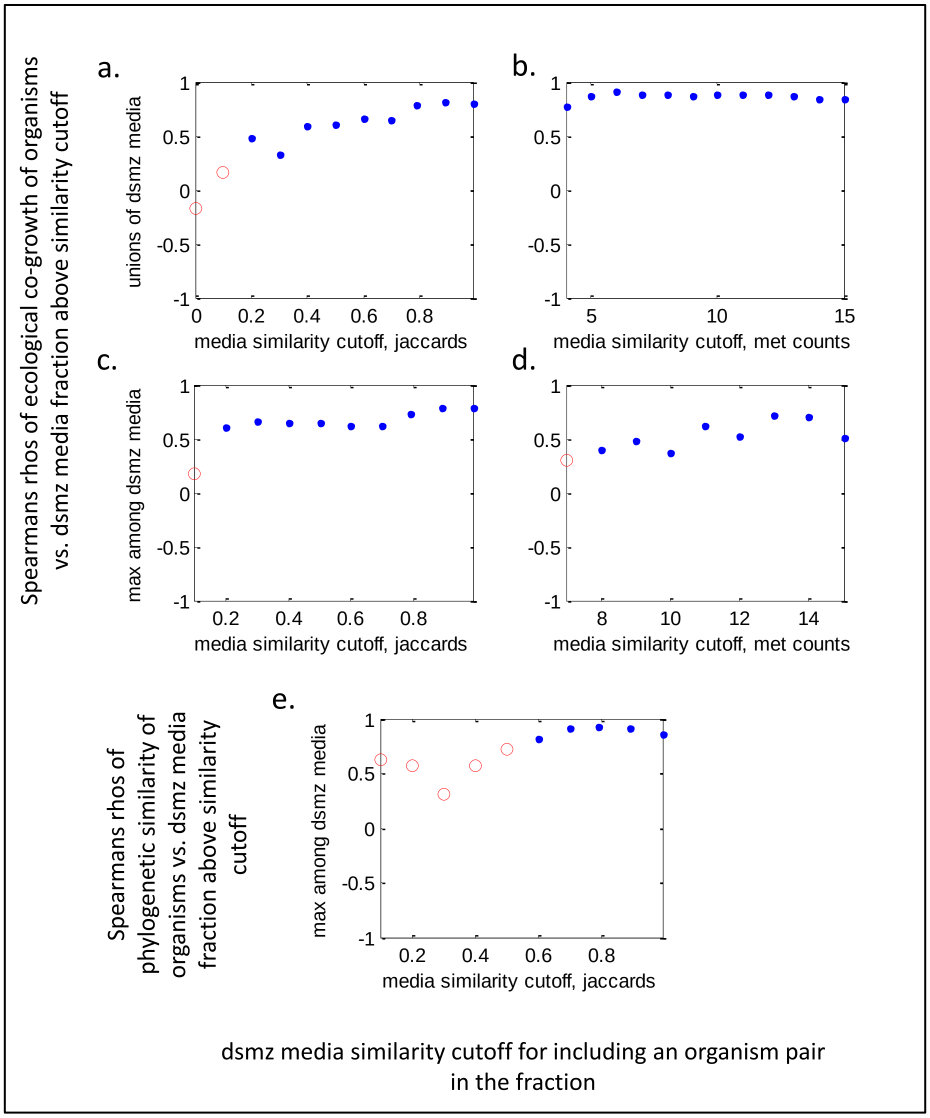

Supplementary Fig. 13. Ecological co-growth vs. sharing of portions of DSMZ media. The figure depicts various types of media similarity cutoffs (x-axis, jaccards in A, C, and E, and overlapping metabolite counts in B and D) vs. (y-axis) the Spearman correlations between the percent of organisms per ecological distance bin (or phylogenetic distance bin, in E) sharing a portion of a medium above the given similarity cutoff (analogous to the correlation coefficients shown in Figure 3).

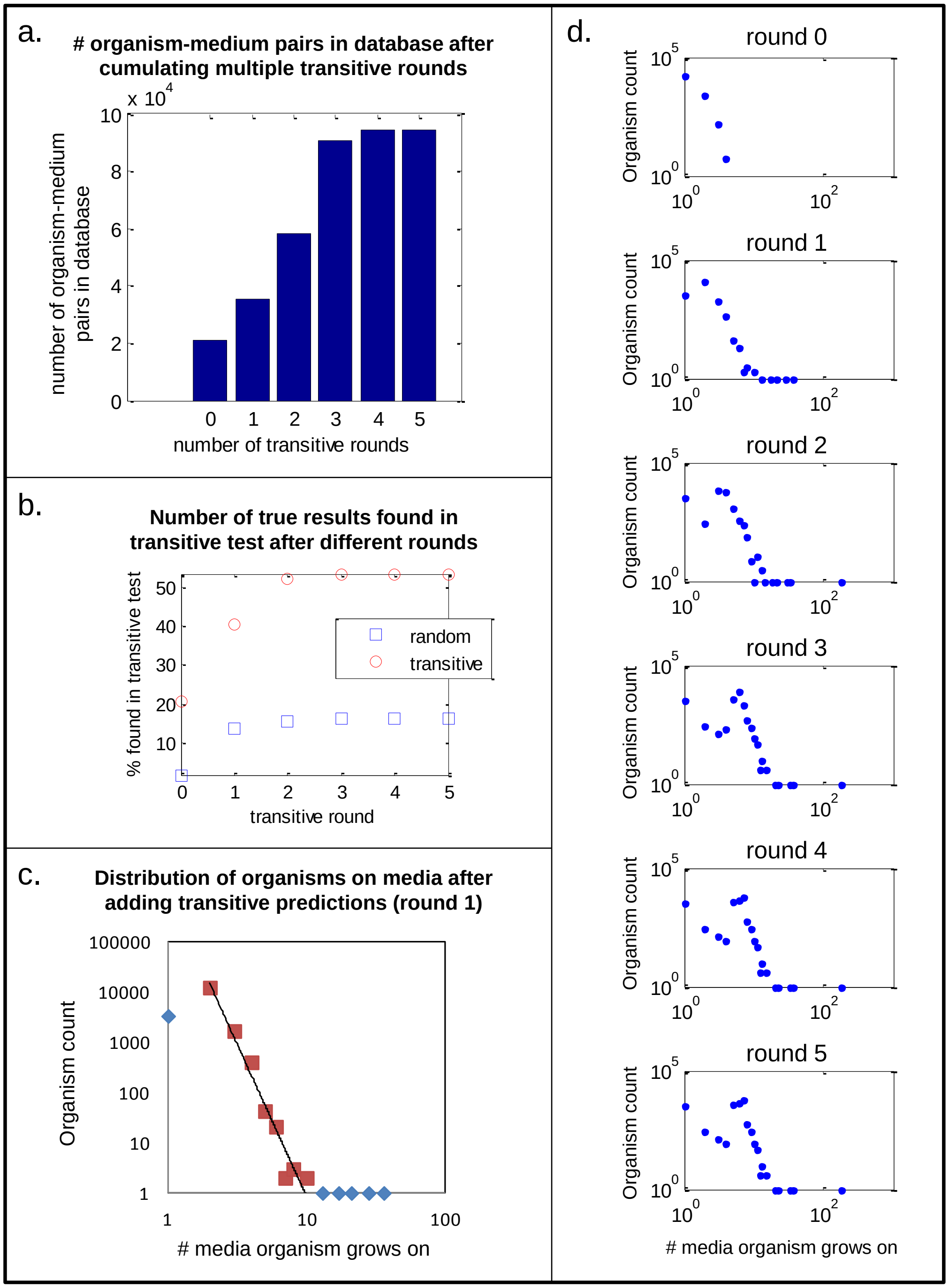

Supplementary Fig. 14. Multiple rounds of transitive prediction. Here we explore the consequences of performing multiple sequential rounds of transitive predictions, adding the predicted org-media pairings into the database after each round. The total number of phenotypes in the database levels off after ~3 rounds.

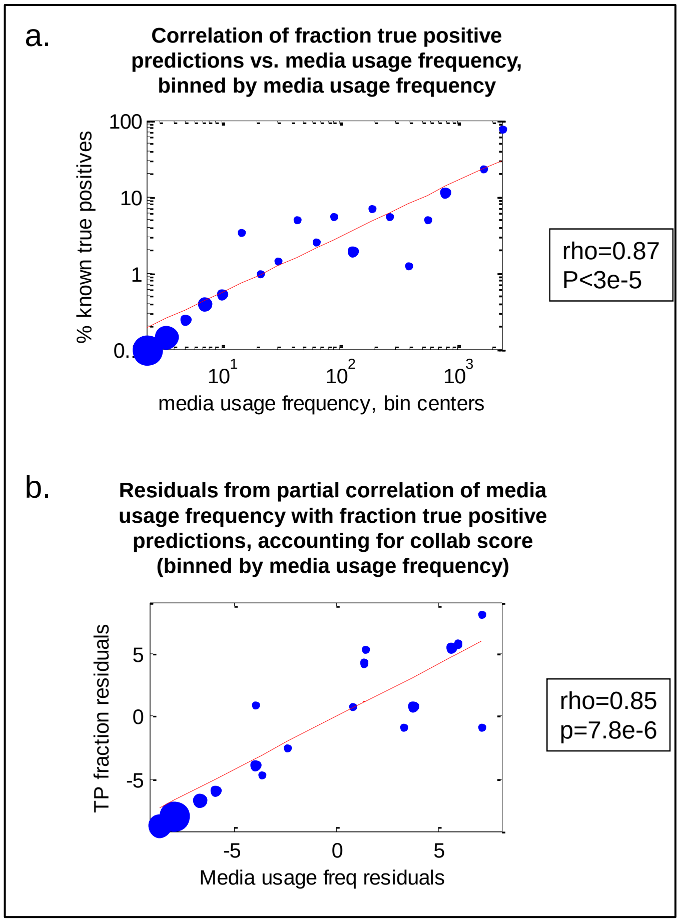

Supplementary Fig. 15. Partial correlation residuals from collab score vs. media usage frequency vs. fraction true positives. (a) correlation of the true positive fraction predicted by collaborative filtering (using the standard GROWREC phylogenetic-based predictor) versus media usage frequency, binned into media usage frequency bins (e.g., instead of binning into collab score bins, as done in Figure 5b of the main text). (b) residuals for partial spearman correlation of media usage frequency vs. fraction of true positive predictions per bin, accounting for collab score (compare these figures to analogous Figure 5b-c).

complex_weights:

The 'weight' is a multiplier on the concentration of the complex component in order to determine how many grams of 'richness' come from it per gram of the component, for determining the rich contribution in media.

complex component	weights
DNA	1
blood	1
casamino	1
charcoal	0
lipid	1
meat	1
peptone	1
plant	1
rumen	1
seawater	0
serum	1

exceptions (by manual curation):

complex_category	component	weight	comment
plant	Air-dried garden soil	0	not very rich
plant	garden soil (with high content of organic matter)	0.1	not very rich
plant	soil extract	0	not very rich
plant	Tea (leaves, black)	0	not very rich
plant	White table wine	0.1	not very rich
seawater	Marine Broth	1	marine broth is a rich component
plant	Oat flakes	0.5	based on comments from Sabine & Elke on media richness (rolled oats is only half richness, so we assigned all 'oats' to 0.5)
plant	Oatmeal	0.5	
plant	Rolled oats	0.5	
rumen	sludge fluid	0.1	based on comments from Sabine & Elke on media richness (sludge fluid is not that rich)
rumen	sludge from an anaerobic digester	0.1	

rich medium cutoffs:

The cutoff is the amount of 'richness' a medium needs to be classed as low, medium, or high 'richness'. Richness is determined by taking a weighted sum of complex components per medium, with weightings described in the table to the left. Also included in 'richness' are certain defined compounds: sugars, sugar-alcohols, and polysaccharides.

boundary	cutoff (units of g/l)
low/mid	5
mid/high	15

saltiness_criteria:

This salt filter is imposed on the collaborative filtering results, and mismatches (organisms only growing on 'low' environments are called 'low' organisms. orgs only growing on 'high salt' environments are called 'salty' organisms. all collaborative filtering predictions that mismatch these, e.g., put a low org with a high salt medium or vice versa, are eliminated.

- Medium 514 salt content (NaCl) is a standard for 'hi salt' orgs..
- We categorize orgs into 'hi' or 'low' salt -- if NaCl content is >=15g/l, its 'high'.
- Also, if a medium contains the word 'sea' anywhere (e.g., artificial seawater, natural seawater, etc.), it is also a high salt medium.

Supplementary Table 1. Weights and cutoffs.

Supplementary Note 1. Future work to be done in KOMODO database curation

Several tasks in reconstructing the KOMODO database were not completed, since they were considerably more complicated than the rest, were not necessary for most of the analyses we intended to perform, or both. We have listed some of these tasks here as a courtesy for future curators and researchers working with the database:

(1) Deal properly with the concentrations of phosphate and other compounds that come from buffers, including phosphate concentrations.
(2) Deal with compounds that are produced in the process of building media (such as sulfur derivatives).
(3) Fix / normalize the gas phase of media.
(4) Incorporate the properties of chelating and reducing agents (e.g., sodium dithionate).
(5) Figure out redox state, ionic strength, of media.
(6) Create special media categories for auxotrophic vs. heterotrophic growth.
(7) Add atmosphere tag to all of the defined media.
(8) Mark complexity of the 'water' type added (to account for potential trace compounds)

Supplementary Note 2. Exploring the media-components matrix via PCA

To gain an overall picture of the compounds that most differentiate known laboratory media, we performed a principle components analysis (PCA) on a compounds-media matrix containing all SEED compound names and molar concentrations within each medium. The first few principle components hold the majority of information in the media (Supplementary Fig. 2a). Compounds that contribute most strongly to these principal components (PCs) are Na^+ and Cl^-, Elemental Sulfur, and HCO_3 (Supplementary Fig. 2b). PC1 in particular holds over 90% of the information in the matrix, and it is dominated by the presence of NaCl. Indeed, media designed for high salt organisms tend to have higher weight in the first principal axis than other media (p=1.6e-31 in ranksum test). Because salt was so dominant, we redid the PCA after removing Na+ and Cl- from the matrix (see Supplementary Fig. 2c). This revealed another small set of compounds that are dominant. Further removals of dominant compounds continued to reveal principle components dominated by compounds that have very high concentration values compared to most compounds in the media matrix. To eliminate these effects, we also performed PCA after setting all concentration values to "1" (Supplementary Fig. 2d). In this case, the loadings of compounds in the first three PCs became highly diffuse. This indicates that a more

detailed analysis is called for in order to gain insight into important compounds across the media (we do this analysis in, Supplementary Note 3, with our analysis across phylogenies).

We next decomposed all SEED compounds into their elemental constituents and performed an additional PCA on an elements-media matrix for all fully defined media. After eliminating variants of a medium with extremely high carbon content (SRB-psychrophile medium), we obtained principle components heavy in Na and Cl (PC1), and H, O, and C (PC2), which together contain 93% of the information in the matrix, thus upholding the general trend seen in the original PCA. Results of the elements-wise PCA are depicted in Supplementary Fig. 3.

Supplementary Note 3. Exploring the components in media used across the tree of life

An important question that can now be asked using KOMODO is how different media components are used across the tree of life. To address this, we built matrices that map enrichment of different phylogenetic groups for media components or component categories. These enrichment matrices were built by performing a 1-sided Fisher's exact test for enrichment of the given component in the given phylogenetic group, as compared to how often it appears across all phylogenetic groups. For calculating the Fisher's exact metric, the number of appearances of a component is counted as the number of bacterial and archaeal strains that are listed as growing on any media containing the compound (e.g., if a phylogenetic group contains 10 strains, 9 of which are listed on media containing Sulfate, then Sulfate would be counted as appearing 9 times of a possible 10 within the group). For ease of visualization and analysis, the final enrichment matrix was built by taking:

$\text{Enrichment} = (-\log10(\text{p-value}))^{\gamma}$,

where the p-values are those calculated as described through the Fisher's exact test, and $\gamma=0.3$. The scaling of the p-values is done to increase their separation from each other and normalize their distribution for visualization and analysis. As shown in the equation above, the scaling consists of two steps: taking the negative log of the p-values, and then applying a gamma correction (γ) (this further spreads the values out, while keeping their order).

We first examined enrichment matrices at the level of Taxonomic Order, looking both across component categories and all individual media components (see Supplementary Fig. 4 and Supplementary Fig. 5, respectively). When viewed at the level of individual components (Supplementary Fig. 5), the taxa clustered into three groups, one of which characteristically contained all Orders in the taxonomic classes Clostridia, Methanomicrobia, and Deltaproteobacteria (with the exception of the Order Myxococcales, which grouped in another cluster). This cluster is characterized by containing a suite of trace metal ions as well as vitamins and cofactors. It is not surprising that these compounds appear together, since they are typically added to media in Elemental mixes and Vitamin mixtures, which contain many trace metals / vitamins at once (depending on the mix). Nevertheless, the relationship between these Orders and

these nutrients might be indicative of a lifestyle factor to be taken in consideration for culturing newly discovered members.

Next, we examined an enrichment matrix of all genera versus media components (see Supplementary Fig. 6; unlike Supplementary Fig. 4 and Supplementary Fig. 5, phyla are clustered here based on the standard taxonomic tree). It is clear from viewing this matrix that there exists an enormous variety of media conditions within any given taxonomic grouping, which reflects the enormous diversity of nutritional capabilities even among related species. As a guide for interested researchers, we have also included a prevalence matrix (Supplementary Fig. 7), in the same format as Supplementary Fig. 6 (i.e., the same organisms and components on the x- and y- axes). This shows the percent of media within a given genus containing each individual component (as opposed to the enrichment matrices, which show only those that are higher in a given genus than among most genera). As expected (i.e., from Figure 2f), the complex-yeast/peptone group comes up as dominant among the most genera.

It is important to note that the DSMZ database, and hence KOMODO, contains the media that organisms are typically grown on, but this does not mean that every component in the medium is necessary or important for growth. So, for example, the enrichment matrices in Supplementary Fig. 5 and Supplementary Fig. 6 show bias for certain non-biologically important compounds among taxonomic groups, such as Myxococcales and Lactobacillales, that contain many members growing on a single medium. Myxococcales, for example, contains 1548 strains in our database, >98% of which grow on a single medium (DSMZ medium 9). Hence, this order is highly enriched for the ingredients of medium 9, despite some of those components not being "characteristic" for, or especially needed by, Mycococcales. For example, the ions sulfate and chloride are present in Medium 9 simply as anions to complement Mg2+, Ca2+ and K+, and are not important for this phylogenetic group in and of themselves. On the other hand, sodium chloride is indispensable for marine organisms, and sulfate is needed as the indispensable electron acceptor in some species. These nuances are difficult to decipher without expert knowledge, but the analyses we present here are a first step towards elucidating them on a large scale. More expansive growth experiments, with many more organism-medium pairings tried and added to the database, will certainly decrease the bias towards such unimportant factors in the future.

To get an overall picture of how phyla cluster at different levels and what compounds tend to separate the growth conditions among them, we clustered phyla-vs.-component enrichment matrices at multiple phylogenetic levels. We then performed PCA on the enrichment matrix at each level (see Supplementary Fig. 8a-f). Next, we examined the compounds that are most dominant in the first PC at each level (Supplementary Fig. 8g-h). A high weight in one of these PCs means that a compound is highly differentiating between taxonomic groups. We found that the compounds filling this first PC (which contains ~25% of the information in the enrichment matrix at various taxonomic levels – see subplot at bottom of Supplementary Fig. 8h) are more concentrated in a few compounds as the taxonomic level becomes more specific (e.g., between Phylum and Genus). This makes sense, and reinforces a view that specific media rules are best determined at the lowest level possible.

The predominance of metal ions in the Order through Genus levels is not surprising, since many such ions are often added to media at once using trace element mixtures. Removal of these mixtures from the media removes these as key compounds in this analysis. Note, this does not detract from the result presented in Figure 2f; there, the assessment is whether compounds are differentiating within Genera; here, we assess whether they are differentiating between Genera. The analysis presented here is more likely to be biased by the existence of trace element mixtures, since each compound is considered a separate dimension for PCA, and all dimensions representing trace elements act as a correlated block that can skew PCA outcomes. In the within-Genera analysis presented in the main paper, there is no PCA done and each compound is treated separately, so this bias should not be an issue.

Supplementary Note 4. The relationship between phylogeny/ecological closeness and media sharing after variations of KOMODO

We were interested in understanding how the correlations between phylogenetic (or ecological) similarity and chance of two organisms to share a lab medium would be affected if we augmented KOMODO with the ~15,000 first-round transitive organism-media predictions. Therefore, we redid the correlations shown in main text Figure 3 after adding the transitive predictions to KOMODO (see Supplementary Fig. 9a-b). For ecological similarity vs. sharing of lab media, a significant correlation was maintained (rho=0.34, p=4.5e-3; see Supplementary Fig. 9b). For phylogenetic similarity, the significance of the correlation was lost, likely due to there being too few phylogenetic distance bins to plot (the correlation was Spearman rho=0.6, p=0.1; see Supplementary Fig. 9a). The numbers of matches are higher overall, which is to be expected, since the KOMODO+transitive database has many more entries in it to match with than KOMODO itself. However, the fact that the correlations go down versus when they are calculated for KOMODO indicates that some of the transitive predictions are likely adding error.

We additionally were interested in whether the same correlations would be upheld if we removed all non-defined media from KOMODO, assessing sharing by organisms of only defined media. To test this, we rerun our analysis of media sharing by organisms of varying phylogenetic or ecological distances, using only fully defined media, instead of using all media in KOMODO as reported main Figure 3 (see Supplementary Fig. 9c-d). One consequence of removing non-defined media is that there are far fewer media and organism-media pairings left in the database. Indeed, there are too few distinct phylogenetic distances left to see a reliable correlation between org-org pair phylogenetic similarity and fraction of org-org pairs sharing a lab medium (only four discrete phylogenetic distance groups remain – see Supplementary Fig. 9c). However, in the ecological space sufficient different ecological distances are mapped that a correlation can be drawn, and we indeed see that the correlation of ecological similarity of organism pairs versus the fraction of pairs sharing a lab medium is yet highly significant (Spearman correlation: rho=0.68, p=9.8e-3, as compared with rho=0.76, p=2.3e-13 using the full KOMODO database; see Supplementary Fig. 9d). We expect that with higher resolution phylogenetic data, the correlation would remain and might even improve when looking only at defined media.

Supplementary Note 5. Media richness and complexity in transitive predictions

It was important to determine how biased transitive predictions are towards rich and/or complex media (complex media are those that contain non-defined components such as yeast extract). To do this, we produced a list of 'transitive triangles,' which are sets of 3 organisms and 3 media that form the training set (equivalent to the schema shown in Figure 4a) and the transitive prediction (the top right box in Figure 4a), examining only transitive predictions where media 1, 2, and 3 are all different media (see schematic in Figure 4a). As expected, we found that rich media turn up more often in these triangles than other media. However, there are particular patterns that interestingly have higher true positive rates than others. The most successful patterns are those in which medium 1 and medium 3 are both rich (as a reminder, the schema is that orgsA&B grow on medium 1, orgsB&C grow on medium 2, and orgC grows on medium 3; given this schema, the transitive prediction is that orgA will grow on medium 3. This is as shown in Figure 4a). This indicates that new rich media can be found for organisms known to grow on rich media, through transitive associations that link through media of any richness. We also examined whether the presence of complex compounds in a medium affects the transitivity. We found that more transitivity occurs among undefined than defined media (i.e., those containing complex components). However, 20% of the predictions include at least one medium that is non-complex.

Supplementary Note 6. Choosing a phylogenetic distance threshold for GROWREC

We tested GROWREC by performing a leave-one-out analysis to compare predicted versus known organism-medium pairings from KOMODO (see Methods). We examined how various thresholds affect the precision of the predictor, and based on this we chose a phylogenetic distance cutoff in the predictor (cutoff was set at a normalized phylogenetic subtree distance of 0.04, with a weighting on collaborative score taken as cutoff / subtree distance; see Supplementary Fig. 10 and Supplementary Fig. 11, and Methods).

To understand better how this threshold affected accuracy of the predictors, we performed a detailed analysis of the fraction of true positives predicted versus the phylogenetic distance cutoff used (we also examined cutoffs using an ecological distance based collaborative filtering method – see Supplementary Fig. 12). We compare the total fraction and total number of true positives predicted as a function of phylogenetic cutoff (Supplementary Fig. 12c-d), as well as the fraction of true positives within the top 10% of score bins (Supplementary Fig. 12e). The cutoff we chose for the paper (0.04) optimizes this latter fraction; however, different cutoffs can be used to optimize results differently (e.g., to get more predictions at the expense of sensitivity, or vice versa) based on these plots. There is no definite cutoff where the relationship becomes invalid, but it does drop significantly above a cutoff of 0.04 and again above a cutoff of 0.13 (see Supplementary Fig. 12c).

Supplementary Note 7. Refining the collaborative filtering predictor based on biologically selective filters

Many factors play into the ability of an organism to grow on a particular medium. Among the most important are the salt content (as shown in Supplementary Fig. 2) and the presence or absence of oxygen, either of which can make an otherwise suitable medium inappropriate for growing a particular strain or species. We therefore sought to incorporate these biological features into our collaborative predictor so that we could eliminate predicting yet untenable cases, such as an aerobic, high salt environment for an anaerobic organism that cannot tolerate salt.

To do this, we developed a method for classifying organisms into usage groups, based on observed patterns of growth on DSMZ media. We utilized for this a tag we had put on each medium when building KOMODO that denotes whether the medium is aerobic or anaerobic (see Figure 1). Using these media definitions, we classified organisms as follows: those that are only listed on aerobic media are classed as aerobes; those listed only on anaerobic media are anaerobes; and those listed on some mixture are considered facultative/aerotolerant. The same logic was used to determine salt preference of organisms, with 'high-salt' media defined as those with above 15g/l NaCl or containing any artificial or natural seawater.

We then imposed these filters on results predicted from collaborative filtering, eliminating predictions that are inappropriate because they pair an anaerobic organism with an aerobic medium, an aerobic organism with an anaerobic medium, a salt-loving organism with a low-salt medium, or a salt-intolerant organism with a high-salt medium. These filters raised the average collaborative scores of predictions, as shown in Figure 5d and discussed in the main text. Note, some media can host either aerobic or anaerobic bacteria (such as blood agar, which can be used for strict aerobes or strict anaerobes). In developing aerobic or anaerobic tags for media, we attempted to follow the most common usage. Future updates and uses of the database can relax some of these tags or replace them in cases where they were poorly assigned.

Supplementary Note 8. Prediction of medium richness

Most laboratory media contain complex components such as 'yeast extract' or 'peptone', which are not fully chemically defined and provide a rich range of nutrients. In practical efforts to cultivate microorganisms**,** these nutrient mixes are advantageous, and usually give the highest initial chance of success. However, it is not always clear how 'rich' a medium for a given organism should be. Some organisms are oligotrophic and thus cannot tolerate rich media; others do best in extensively rich nutrient broths.

Because the richness is such a ubiquitous practical consideration in developing media, we leveraged our collaborative filtering tool towards predicting the richness preferences of bacteria. We define medium richness as a weighted sum of complex components such as 'yeast extract' and carbon-providing but defined nutrients such as sugars, polysaccharides, and sugar alcohols (see Supplementary Table 1 and

Supplementary Data 4 for lists of rich compounds and their weightings). We then binned media as low, medium, or high 'richness' using cutoffs of 5g/l and 15g/l of rich nutrients. In these categorizations, we obtained ~92% accuracy vs. manually curated media richness classifications from DSMZ curators, after some optimization.

We next built a GROWREC-based predictor of organism richness preference, which works by summing collaborative scores for all predicted media in each richness category and then choosing the category with the highest collective score. This predictor achieved high accuracy against organism richness preferences manually curated by DSMZ curators and against a 'gold-standard' set of organism richness preferences observed in KOMODO (accuracies are 84.3% and 97.9% over 102 and 430 predictions, respectively, against manual curations and the gold standard -- gold standard was built from cases of organisms known to grow in only one medium richness, as explained in Methods). We also calculated organism richness preferences by taking a weighted mean of richness amounts weighted by collab score, and obtained nearly identical results. We found that as the richness preference of the organism rises, so does the richness of actual media the organisms are known to grow on in KOMODO (Figure 6). These analyses indicate that our method is highly predictive of the richness preferences of organisms, and could be helpful in optimizing new media.

Supplementary References

1. Henry CS, DeJongh M, Best AA, Frybarger PM, Linsay B, Stevens RL. High-throughput generation, optimization and analysis of genome-scale metabolic models. *Nat Biotechnol* **28**, 977-982 (2010).

2. Henry CS*, et al.* Connecting genotype to phenotype in the era of high-throughput sequencing. *Biochim Biophys Acta*, (2011).
